# Quality of DCIS information on the internet: a content analysis

**DOI:** 10.1007/s10549-019-05315-8

**Published:** 2019-06-18

**Authors:** Jayden Blackwood, Frances C. Wright, Nicole J. Look Hong, Anna R. Gagliardi

**Affiliations:** 1 Toronto General Hospital Research Institute, University Health Network, 200 Elizabeth St, Toronto, ON M5G 2C4 Canada; 2 Sunnybrook Health Sciences Centre, 2075 Bayview Ave, Toronto, ON M4N 3M5 Canada

**Keywords:** Ductal carcinoma in situ, Patient-centred care, Internet, Consumer health information, Information tools, Quality assessment

## Abstract

**Purpose:**

Women with ductal carcinoma in situ (DCIS) experience lingering confusion and anxiety, and may use the Internet for supplemental information. This study assessed the content and quality of DCIS information on the Internet.

**Methods:**

We searched Google for English-language, publicly available DCIS information tools published from 2010 to current by non-profit organizations. We summarized tool characteristics, DCIS labels, and information important to women with DCIS corresponding to domains of a patient-centred care (PCC) framework. Tool quality was appraised with the DISCERN instrument.

**Results:**

Of 39 tools included, most were plain language summaries published since 2016. Tools employed a median of 2.0 labels (range 1.0 to 5.0) for DCIS, most frequently non-invasive breast cancer (29, 74.4%), abnormal cells (14, 35.9%), pre-cancer (14, 35.9%), and early form of breast cancer (13, 33.3%). Tools addressed a median of 4.0 (range 2.0 to 5.0) PCC domains. Few tools contained content in the domains of fostering the relationship (30.8%), addressing emotions (41.0%), or follow-up (41.0%); 74.4% noted the risk of progression or recurrence but provided vague details. Tools were assessed as high (25.6%), moderate (48.7%), and low (25.6%) quality.

**Conclusions:**

Few DCIS information tools available to women on the Internet meet quality criteria for consumer health information or address concerns of importance to women with DCIS. By identifying a range of poorly defined terms used to label DCIS, and specific content domains that were lacking, this study identified how existing tools could be improved, and identified higher-quality tools that clinicians can use when discussing DCIS with patients.

**Electronic supplementary material:**

The online version of this article (10.1007/s10549-019-05315-8) contains supplementary material, which is available to authorized users.

## Background

Patient-centred care (PCC), which addresses patient clinical needs, life circumstances, and personal preferences [1, 2], is a fundamental element of high-quality health care because it has improved multiple patient and health system outcomes across numerous settings of care [3–6]. A scoping review of 19 studies published from 1994 to 2011 identified 25 unique frameworks or models that offer insight on how to achieve PCC [7]. Common domains across those PCC frameworks are predicated on information, which facilitates patient–clinician communication, educates and empowers patients, addresses patient concerns or emotions, clarifies uncertainties about risks and outcomes, enables informed decision-making, and enhances patient self-management [8]. Various types of information tools, acquired by patients themselves or provided to patients by clinicians, support PCC. For example, educational material helps patients understand their condition [9], question prompt lists help patients communicate with clinicians [10], and decision aids help patients articulate preferences [11]. Single interventions of information tools in print or electronic format that inform or activate patients can positively impact patient knowledge, communication, decision-making, and health behaviour [12].

PCC is particularly relevant for conditions where there is limited evidence to support decision-making, two or more treatment options are suitable, or when treatment outcomes may be adverse as is sometimes the case for ductal carcinoma in situ (DCIS). DCIS includes several abnormal cell types confined to the breast ducts with variable risk of progression and recurrence [13]. While about 20% of women with DCIS will develop invasive disease, the 20-year breast cancer-specific mortality is a favourable 3.3% [14]. The effectiveness of tests to predict progression [15], and management with active surveillance [16–18] are under investigation. Thus, standard care is lumpectomy or mastectomy and possible radiotherapy or hormone therapy [19, 20], Physicians have said it is difficult to describe DCIS [21, 22]. Women with DCIS told they have “stage 0 cancer” or “pre-cancer”, have inaccurate perceptions of the risk of invasive cancer, recurrence and survival, and report receiving little clarifying information or opportunity for discussion, causing confusion and anxiety well after treatment, and reduced health-related quality of life (HRQoL) [23–26]. Thus, while clinical outcomes may be favourable, the patient experience during and after treatment may be less than ideal.

Clearly, to improve PCC and HRQoL for women with DCIS, tools are needed that offer thorough and accurate information about DCIS, and distinguish it from invasive breast cancer. Increasingly, patients turn to the Internet for health information, and women use the Internet as a source of health information more than men [27]. Sharing of Internet-acquired information by patients during appointments can foster communication and improve the patient–clinician relationship [28], and supplement information provided by clinicians [29]. However, clinicians have raised concerns about the accuracy of online health information [30]. The purpose of this study was to assess the content and quality of DCIS information tools available to women on the Internet. This may identify useful resources that could be broadly employed to support PCC for DCIS, or it may reveal the need to develop or improve DCIS information tools if such resources are sparse or of poor quality.

## Methods

### Approach

Content analysis was employed to assess DCIS information tools identified on the Internet [31]. A manifest content analysis approach was used [32]. This refers to qualitatively and/or quantitatively describing explicit content as reported in written, verbal, or visual communication, without theoretical analysis or interpretation of its underlying meaning. Directed/deductive and summative content analysis techniques were employed to categorize information within tools according to an existing PCC framework (directed/deductive) [8], and to enumerate the number and type of information tools and their content (summative) [31, 32]. While not technically a literature synthesis, the Preferred Reporting Items for Systematic Reviews and Meta-Analyses (PRISMA) criteria guided the conduct and reporting of the review [33]. Ethics review and approval was not necessary because information material was publicly available.

### Eligibility

In the absence of a gold-standard definition or taxonomy of patient information tools, we defined eligible tools using the Workgroup for Intervention Development and Evaluation Research (WIDER) recommendations for reporting knowledge-based interventions (Table 1) [34].

**Table 1:** Inclusion criteria for information tools

Criteria	Description
Recipients	Those who might look for and use DCIS information material including adult women aged 18 or older diagnosed with or treated for DCIS, family members or care partners, or clinicians who care for women with DCIS and wish to provide them with or direct them to information
Personnel or setting	Provided to patients by clinicians, made available to patients by health care organizations (i.e. in physician offices or waiting rooms, clinics, patient libraries, etc.), or acquired by patients in print or online from the developers or other agencies
Developers	Non-profit organizations such as government, governmental agency, academic group, professional society, or disease-specific foundation in Canada, or in English-language countries: Australia, Canada, England, Ireland, New Zealand, Scotland, and the United States
Content	Information about DCIS, or about breast cancer but including information about DCIS, at minimum, naming and defining or explaining DCIS, conveyed by text, numbers or statistics, and/or graphics, including but not limited to DCIS diagnosis, pathology, prognosis, treatment, risks, outcomes, and follow-up care
Format	Including but not limited to plain language summaries, question prompt lists, decision aids, etc. published or updated in 2010 or later to reflect recommendations for DCIS management in the most current clinical guidelines [19, 20]
Delivery	Available on the Internet to be viewed on web pages or online files, or downloaded to print format
Intensity or duration	Could be employed directly before, during, or upon conclusion of a clinical appointment, or viewed online at any time on multiple occasions as desired

Exclusion criteria were generated concurrent with screening. The following types of information tools were not eligible: publication date or developer not specified, requiring subscription or purchase, clinical practice guidelines, research articles or editorials, risk assessment calculators, clinical trials, insurance services, keynote speaker presentations, videos, news items, social media forums or support groups, or Wikipedia or other similar open-editing web sites. Resources developed by for-profit organizations were excluded due to inconsistency in availability and cost. While some of these excluded tools such as videos or support groups may provide women with information about DCIS, we aimed for uniformity in selecting print or electronic tools comprised of text and/or graphics so that quality could be assessed similarly using a common instrument and compared across tools.

### Searching

JB searched the Internet using Google from 22 June to 27 June 2018. With no standard methods for searching “grey literature” on the Internet [35, 36], we employed the following strategy. JB conducted preliminary searches of Google using “DCIS or ductal carcinoma in situ” to develop a list of labels or titles for potential information tools until no further unique terms emerged. Then “DCIS or ductal carcinoma in situ” were successively combined with each of the unique labels for potential information tools including booklet, brochure, decision aid, fact sheet, handout, leaflet, pamphlet, patient information, communication aid, question prompt list, summary, and toolkit. JB and BN conducted a pilot test to determine how many pages of Google search results included relevant results. They executed the same four searches and perused the results to assess how many results were relevant. Both agreed that no further potentially relevant results were apparent after six pages (ten results per page). JB proceeded to search Google using all search term combinations. All search results were exported or copied into an Excel spreadsheet for screening.

### Screening

JB, BN, HL, and ARG independently screened titles of the first 100 search results against eligibility criteria. JB compared independent screening to identify discrepancies, and ARG met with JB, BN, and HL to discuss the discrepancies and how to apply the screening criteria. Then JB screened all remaining titles. All potentially relevant items were retrieved for full-text screening. JB and BN independently screened 50 full-text tools on 10 July 2018. Discrepancies were resolved through discussion with ARG. JB then screened remaining full-text tools.

### Data extraction

A data extraction form was developed in Excel to collect tool attributes (title, author or developer, country, publication year, tool type, breast cancer or DCIS-specific, DCIS definition, and labels) and content. Content extracted from each tool (Table 2) corresponded to the top ten questions deemed essential from among 117 unique questions specified by women with DCIS when asked what they most wanted to know upon diagnosis [37], which we mapped to domains of the McCormack et al. PCC framework [8], chosen because it is the most comprehensive PCC framework and specific to cancer. To pilot data extraction, JB and BN independently extracted data from six tools on 22 Aug 2018 and identified discrepancies, which were discussed with ARG to achieve consensus. JB extracted data from all remaining tools.

**Table 2:** Data extracted from included tools by PCC domain

McCormack et al. PCC framework [8]		Data extracted corresponding to questions prioritized by women with DCIS [37]
Domain	Domain concepts	
Fostering the patient–clinician relationship	• Discuss roles and responsibilities • Honesty and openness • Trust in clinician competence • Express caring • Build rapport	Which doctors should I be seeing and when?
Exchanging information	• Explore needs and preferences • Share information • Provide information resources • Assess and facilitate understanding	How does DCIS differ from invasive cancer?
Addressing emotions	• Explore and identify emotions • Assess anxiety or depression • Validate emotions • Express empathy or reassurance • Provide help to deal with emotions	What services are available to me for support?
Managing uncertainty	• Define uncertainty • Assess uncertainty (cognitive) • Use emotion-focused (affective) and problem-focused (behavioural) management strategies	What is the chance DCIS will turn into invasive cancer? What is the change of DCIS coming back in either breast or spreading to the lymph nodes or to the rest of the body?
Making decisions	• Communicate about decision needs • Prepare for deliberation and decision • Make a choice and action plan • Assess decision quality	What are my treatment options? Which treatment offers the smallest chance of DCIS returning?
Enabling self-management	• Learn and assess • Share and advise • Prioritize and plan • Prepare, implement, and assist • Arrange and follow-up	How should I be followed after treatment for DCIS? What things should I do to prepare for the postoperative period?

### Data analysis

Summary statistics were used to report the number and proportion of tools by type and per content extracted. The quality of all included items was assessed with DISCERN, a validated, widely used instrument to assess the quality of consumer health information, comprised of 15 items pertaining to reliability and content, plus 1 overall quality score [38]. Each item was independently assessed by JB and BN, who achieved consensus through discussion and consultation with ARG, who resolved discrepancies. TF independently confirmed scoring. As per scoring instructions, each items is scored on a 5-point scale converted to yes (5), partially (2–4) or not met (1), and overall scores were based on item scoring.

## Results

### Search results

Searching resulted in 759 unique results, and 621 were excluded by title screening. Among 138 full-text tools screened, 96 were excluded based on publication type (51) or date (11), they could not be located (15), lacked any DCIS content (14), were not freely available (9) or were developed by a for-profit organization (3), leaving 39 tools eligible for review (Supplementary File 1). Extracted data are included in Supplementary File 2 [39–80].

### Tool characteristics

Tool attributes are summarized in Table 3. Information tools were published between 2011 and 2018; nearly two-thirds (27, 69.2%) were published more recently in the last 3 years from 2016 to 2018. Most tools were developed by organizations in the United States (18, 46.2%), followed by England (10, 25.6%), Australia (5, 12.8%), 2 (5.1%) in each of Canada and New Zealand, and 1 (2.6%) in each of Ireland and Scotland. Information tools were largely developed by charities or foundations (13, 33.3%), treatment centres/hospitals (10, 25.6%), and governments (6, 15.4%) or governmental agencies (5, 12.8%), followed by patient support services (2, 5.1%), and 1 (2.6%) each by a professional society, patient advocacy group, and partnership between a charity and an academic institution. With respect to tool type, most tools were printable plain language summaries featuring text and/or graphic information (25, 64.1%, range 1 to 107 pages) that were labelled by developers as booklets, brochures, fact sheets, FAQs, handouts, leaflets, or pamphlets. Remaining tools were web sites (12, 30.8%) comprised of single or multiple pages, and 2 (5.1%) were printable decision aids. Most tools were specific to DCIS (25, 64.1%) while 14 (35.9%) pertained to breast cancer and included information about DCIS.

**Table 3:** DCIS information tool characteristics

Organization (country, date)	Organization type	Title	DCIS-specific	Tool type
Breast Cancer Care UK, England, 2018 [39]	Charity or foundation	Ductal carcinoma in situ (DCIS)	Yes	Plain language summary (16 pages)
Cancer Council Western Australia, Australia, 2018 [40]	Charity or foundation	Understanding breast Cancer	No	Plain language summary (76 pages)
Dr. Susan Love Research Foundation, United States, 2018 [41]	Charity or foundation	Ductal carcinoma in situ (DCIS)	Yes	Web site (multiple screens)
National Comprehensive Cancer Center, United States, 2018 [42]	Treatment centres	Breast cancer non-invasive	No	Plain language summary (50 pages)
National Health Service, Scotland, 2018 [43]	Government	Informing women about the benefits and risks of breast screening	No	Plain language summary (3 pages)
Cancer Care Nova Scotia, Canada, 2018 [44]	Government agency	Information for patients receiving radiation therapy: Breast cancer or ductal carcinoma in situ (DCIS) of the breast	No	Plain language summary (8 pages)
Susan G. Komen, United States, 2018 [45]	Charity or foundation	Facts for life: ductal carcinoma in situ	Yes	Plain language summary (2 pages)
Susan G. Komen, United States, 2018 [46]	Charity or foundation	Treatment for DCIS	Yes	Web site (single screen)
Ohio State University Comprehensive Cancer Center, United States, 2018 [47]	Treatment centre	Ductal carcinoma in situ (DCIS) of the breast	Yes	Plain language summary (3 pages)
The Pennine Acute Hospitals, England, 2018 [48]	Treatment centres	Enhanced recovery after breast surgery: an information guide	No	Plain language summary (28 pages)
University of Iowa Hospitals and Clinics, United States, 2018 [49]	Treatment centre	Ductal carcinoma in situ (DCIS)	Yes	Web site (single screen)
American Society of Clinical Oncology, United States, 2017 [50]	Professional society	ASCO answers: breast cancer	No	Plain language summary (2 pages)
BreastCancer.org, United States, 2017 [51]	Charity or foundation	Ductal carcinoma in situ (DCIS)	Yes	Web site (multiple screens)
Breast Screen Aotearoa, New Zealand, 2017 [52]	Government agency	Breast conditions: ductal carcinoma in situ	Yes	Plain language summary (2 pages)
Cancer Australia, Australia, 2017 [53]	Government agency	Ductal carcinoma in situ	Yes	Web site (single screen)
Cancer Research UK, England, 2017 [54]	Charity or foundation	Ductal carcinoma in situ (DCIS)	Yes	Web site (single screen)
Cancer Treatment Centers of America, United States, 2017 [55]	Treatment centres	Ductal carcinoma in situ	Yes	Web site (single screen)
Health Talk.org, University of Oxford and DIPEx, England, 2017 [56]	Charity or foundation, Academic partnership	Ductal carcinoma in situ (DCIS)	Yes	Web site (multiple screens)
National Health Service, England, 2017 [57]	Government	NHS breast screening: Helping you decide	No	Decision aid (16 pages)
Alaska Breast Care and Surgery, United States, 2016[58]	Treatment centre	DCIS—Ductal carcinoma in situ	Yes	Web site (single screen)
American Cancer Society, United States, 2016 [59]	Charity or foundation	Treatment of ductal carcinoma in situ (DCIS)	Yes	Web site (single screen)
California Department of Health Care Services, United States, 2016 [60]	Government	A woman’s guide to breast cancer treatment	No	Plain language summary (48 pages)
Living Beyond Breast Cancer, United States, 2016 [61]	Patient support service	Guide for the newly diagnosed	No	Plain language summary (60 pages)
National Health Service, England, 2016 [62]	Government	Breast cancer in women	No	Web site (multiple screens)
Worcester Breast Surgery, England, 2016 [63]	Treatment centre	Ductal carcinoma in situ (DCIS)	Yes	Plain language summary (1 page)
The Newcastle upon Tyne Hospitals, England, 2016 [64]	Treatment centres	Ductal carcinoma in situ treatment completion	Yes	Plain language summary (2 pages)
Princess Margaret Hospital – University Health Network, Canada, 2016 [65]	Treatment centre	Know about ductal carcinoma in situ (DCIS) and treatment	Yes	Plain language summary (4 pages)
American Cancer Society, United States, 2015 [66]	Charity or foundation	Special section: Breast carcinoma in situ	Yes	Plain language summary (11 pages)
Macmillan Cancer Support, England, 2015 [67]	Patient support service	Understanding ductal carcinoma in situ (DCIS)	Yes	Plain language summary (107 pages)
Westmead Breast Cancer Institute, Australia, 2015 [68]	Treatment centre	Ductal carcinoma in situ (DCIS)	Yes	Plain language summary (2 pages)
Breast Cancer Action, United States, 2014 [69]	Advocacy group	Ductal carcinoma in situ (DCIS)	Yes	Plain language summary (2 pages)
Breast Cancer Now, England, 2013 [70]	Charity or foundation	The best treatment: your guide to breast cancer treatment in England and Wales	No	Plain language summary (70 pages)
Cancer Australia, Australia, 2013 [71]	Government agency	Breast cancer fact sheet	No	Plain language summary (2 pages)
Irish Cancer Society, Ireland, 2013 [72]	Charity or foundation	Ductal carcinoma in situ (DCIS)	Yes	Plain language summary (4 pages)
National Cancer Institute, United States, 2012 [73]	Government	About this booklet: medical care for women with breast cancer	No	Plain language summary (32 pages)
National Cancer Institute, United States, 2012 [74]	Government	Surgery choices for women with DCIS or breast Cancer	No	Decision aid (24 pages)
HealthDirect, Australia, 2012 [75]	Government agency	Breast cancer: Pre-invasive ductal carcinoma	Yes	Web site (single screen)
Cancer Prevention and Treatment Fund, United States, 2011 [76]	Charity or foundation	DCIS: What you need to know	Yes	Plain language summary (18 pages)
Cancer Society NZ, New Zealand, 2011 [77]	Charity or foundation	Ductal carcinoma in situ	Yes	Plain language summary (3 pages)

### DCIS labels

Tools employed a median of 2.0 labels (range 1.0 to 5.0) for DCIS (Supplementary File 3). The most frequently used term was non-invasive breast cancer (29, 74.4%). Approximately one-third of tools employed the terms abnormal cells (14, 35.9%), pre-cancer or pre-invasive cancer (14, 35.9%), and early form of breast cancer (13, 33.3%). Fewer tools used the labels stage 0 cancer (9, 23.1%) or cancer cells (6, 15.4%). Only 2 (5.1%) tools solely used the term ductal carcinoma in situ, and only 1 (2.4%) tool specified that it was not breast cancer.

### PCC domains

No tools addressed all six PCC domains [7] corresponding to questions of importance to women with DCIS [37] (Supplementary File 4). Tools addressed a median of 4.0 PCC domains (range 2.0 to 5.0). A total of 10 (25.6%), 10 (25.6%), 17 (43.6%), and 2 (5.1%) tools addressed 5, 4, 3, and 2 PCC domains, respectively. Findings did not appear to differ by publication year, country, or developer.

#### Fostering the relationship

Fostering the relationship was the least-addressed domain. A total of 12 (30.8%) tools named specialties involved in DCIS care. For example: “Surgical, medical and radiation oncologists work together with pathologists, plastic and reconstructive surgeons, nurses, genetics counselors and pharmacists to develop an individualized treatment plan…” Ten (25.6%) tools also specified the role of each specialty. For example: “The pathologist writes a pathology report that says whether you have DCIS or not”. and “A radiation oncologist is a doctor who gives breast cancer patients special X-rays to kill their cancer cells”.

#### Exchanging information

Most tools addressed “exchanging information” by defining or describing DCIS. A total of 37 (94.9%) specified that DCIS was distinct from invasive breast cancer because it was contained within the milk ducts. For example: “DCIS is a non-invasive type of breast cancer that occurs when abnormal cells are found solely within the milk ducts”. A total of 12 (30.8%) also described different grades of DCIS. For example: “With ductal carcinoma in situ (DCIS) the three grades are usually called low, intermediate and high instead of 1, 2 or 3”. Four (10.3%) tools also included questions that patients could ask of clinicians.

#### Addressing emotions

Resources inconsistently “addressed emotions” by acknowledging or validating feelings of concern or anxiety (16, 41.0%) or offering coping strategies (7, 17.9%), and this content was often brief or vague. For example, “Finding out you have breast cancer can leave you feeling a range of emotions. Fear, shock, sadness and anger are all common feelings at this time. Remember that there are people who can support you so do not be afraid to ask for help”. [72]

#### Managing uncertainty

Many tools addressed “managing uncertainty” by noting the risk of progression or recurrence (29, 74.4%). However, most provided brief and vague information; for example: “DCIS has a very good prognosis (outlook)” [39] or “Risk of invasive cancer or DCIS recurrence is very low”. [60] Only 8 (20.5%) tools offered statistics to describe risks; for example, “Most recurrences happen within 5–10 years of initial diagnosis. The chances of recurrence are under 30%” [51] or “Women who have had DCIS are 4–12 times more likely to develop subsequent invasive breast cancer despite treatment”. [75]

#### Making decisions

All 39 tools addressed “making decisions” by describing treatment options including different means of diagnosis, surgical approaches, adjuvant treatments, and options for reconstruction following mastectomy. Of those, 26 (66.7%) tools also rationalized the need for treatment despite DCIS being non-invasive. For example: “DCIS may, if left untreated, develop the ability to spread outside of the ducts, known as invasive cancer”. [48]

#### Enabling self-management

Enabling self-management was inconsistently addressed by included tools by describing what to expect after treatment. Few tools described follow-up care (16, 41.0%) and most of those provided little detail; for example, “Now that you have been diagnosed with DCIS, it is very important that you have regular breast exams”. [76] A greater number of tools (24, 61.5%) offered self-care advice with respect to lifestyle factors and/or provided contact information or links for sources of additional information or support.

### Quality assessment

With respect to quality, 10 (25.6%), 19 (48.7%), and 10 (25.6%) tools were assessed with the DISCERN instrument [38] as high, moderate, and low quality, respectively (Supplementary File 5). High quality was not associated with year of publication, country, or developer type; however, 6 of 10 (60.0%) high-quality tools addressed 5 of 6 PCC domains.

## Discussion

This study identified 42 DCIS information tools available to women on the Internet. Most were printable plain language summaries published since 2016 that referred to DCIS using a wide variety of labels. Many tools failed to address PCC domains that correspond to questions of importance to women with DCIS [8, 37], and few tools were assessed as high quality [38]. Overall, these findings underscore the need to develop or improve DCIS information tools that could help clinicians communicate with women diagnosed with DCIS, and supplement clinician discussions to reduce confusion and inaccurate perceptions among women.

Other research highlights increasing use of the Internet as a source of breast cancer information, and the poor quality of that information. For example, analysis of Google data revealed increasing use of the Internet for cancer information, and that breast cancer was the top search [78]. Self-report questionnaire data from 27,491 breast cancer patients between 2007 and 2013 also showed increasing use of the Internet as a source of information, particularly among those younger than 70 years of age, with 10 or more years of formal education, and lower cancer stage [79]. Assessment of 30 English- and 30 German-language web sites on side effects of radiotherapy for breast cancer with DISCERN found that most were poor quality [80]. While Lo et al. [37] identified questions important to women with DCIS, we found no prior published studies that developed or evaluated DCIS information tools. Similarly, in a scoping review of 51 studies published from 1997 to 2016, we identified only two that developed or evaluated educational interventions targeted to either patients or clinicians to support patient awareness, knowledge, discussions, or decision-making about DCIS [81]. Thus, our findings are unique in their focus on DCIS, and in the context of related research demonstrating increasing use of the Internet as a source of information by breast cancer patients, emphasizes the importance of developing or improving DCIS information tools on the Internet.

Our research identifies specific ways to develop, or improve the content and quality of DCIS information tools. We found that DCIS information tools used a wide variety of terms, most commonly, non-invasive breast cancer, abnormal cells, pre-cancer or pre-invasive cancer, or early form of breast cancer; thus, one option is to consistently employ and define terms for DCIS. It is well-recognized that use of medicalized terms by clinicians prompts higher anxiety ratings and perceived disease severity among patients with various conditions, and preference for more aggressive management [82, 83]. This is also true of women with DCIS, who preferred use of terminology without the cancer label to describe DCIS [84, 85]. While some experts have advocated for changes to terminology for lesions with low malignant potential [86], other experts argue that use of non-cancer labels may be deceptive and limit informed decision-making [87]. Using a two-round Delphi of 27 women with DCIS and 29 clinicians, we generated Canadian consensus recommendations on PCC for DCIS, which included a recommendation to refine DCIS nomenclature [88], In future research, we plan to engage international experts including DCIS survivors, clinicians, cancer staging and classification agencies, cancer societies, and breast cancer trials groups and advocacy groups in identifying the potential benefits and harms of various DCIS labels.

A second option for improving the content and quality of DCIS information tools is to more thoroughly address PCC domains, which correspond to questions of importance to women with DCIS [8, 37]. Doing so may help clinicians discuss DCIS with patients, engage patients in their own care, and improve the care experience and HRQoL in women newly diagnosed with DCIS and DCIS survivors. In particular, our study found that few tools contained content relevant to the domains of fostering the relationship, addressing emotions, managing uncertainty, or enabling self-management, demonstrating the need for DCIS tools to include more information on the role of various specialties involved in DCIS care; acknowledging and validating feelings of concern or anxiety, and offering coping strategies; providing specific details about the risk of progression to invasive breast cancer and of recurrence; and describing follow-up care processes and self-care advice. Once developed, research is needed to evaluate the impact of such tools on patient–clinician communication and patient outcomes such as perceived PCC and HRQoL.

With respect to clinical practice, these findings could help clinicians to direct women with DCIS to useful online tools since patients who conduct their own searches may not find all resources that were assessed as high quality or appraise the resources they find to ascertain those that might be more useful. Also, clinicians could benefit from reviewing the resources assessed as high quality to help them in discussion DCIS with their patients.

Strengths of this study include use of rigorous and unique methods to identify and screen tools, and extract and summarize data; compliance with research reporting standards [33]; use of an established PCC framework [8], which corresponds to the concerns of women with DCIS [37], upon which to map DCIS information tool content; and use of a validated instrument to assess DCIS information tool quality [38]. Several factors may limit the interpretation and application of the findings. Despite employing a thorough search strategy, we may not have identified all relevant DCIS information tools, particularly because we restricted our search to English-language tools and resources developed only by non-profit organizations. Furthermore, we did not include for-profit Internet resources that may be available to and used by women.

In conclusion, despite increasing use of the Internet as a source of information by women with breast cancer or DCIS, only 25.0% of DCIS information tools available to women on the Internet meet quality criteria for consumer health information or address concerns of importance to women with DCIS. By identifying a range of poorly defined terms used to label DCIS, and specific content domains that were lacking, this study revealed how existing tools could be improved, and important considerations for the future development of tools, and identified resources that clinicians can use when discussing DCIS with patients.

## Electronic supplementary material

Below is the link to the electronic supplementary material.

Supplementary material 1 (DOCX 19 kb)

Supplementary material 2 (DOCX 87 kb)

Supplementary material 3 (DOCX 18 kb)

Supplementary material 4 (DOCX 16 kb)

Supplementary material 5 (DOCX 19 kb)
