# Supplementary material for: Quality of DCIS information on the internet: a content analysis
Source: Breast Cancer Res Treat. 2019 Jun 18;177(2):295–305. doi: 10.1007/s10549-019-05315-8 (PMC6661062; doi:10.1007/s10549-019-05315-8)
Supplement: Supplementary file 1 — Supplementary material 1 (DOCX 19 kb) [file 10549_2019_5315_MOESM1_ESM.docx]

Supplementary File 1. PRISMA diagram

Google

n = 1354

Records after duplicates removed

n = 759

Full-text items screened

n = 138

Titles/abstracts excluded

n = 621

Items included in content analysis

n = 39

Full text items excluded

n = 99

- Publication type ineligible (47)
- Not found (15)
- No DCIS content (14)
- Not freely available (9)
- Publication date unknown or before 2010 (11)
- For-profit organization (3)
