# Supplementary material for: Quality of DCIS information on the internet: a content analysis
Source: Breast Cancer Res Treat. 2019 Jun 18;177(2):295–305. doi: 10.1007/s10549-019-05315-8 (PMC6661062; doi:10.1007/s10549-019-05315-8)
Supplement: Supplementary file 2 — Supplementary material 2 (DOCX 87 kb) [file 10549_2019_5315_MOESM2_ESM.docx]

Supplementary File 2. Data extracted from included DCIS information tools

| Organization | Specialty role | DCIS labels | DCIS description | Rationale for treatment | Anxiety | Risk of progression or recurrence | Prognosis | Prognosis statistics | Treatment | Follow-up and self-care | Other tools or support |
| --- | --- | --- | --- | --- | --- | --- | --- | --- | --- | --- | --- |
| Breast Cancer Care UK | NR | early form of breast cancer, pre-invasive, intra-ductal, non-invasive cancer, or Stage 0 breast cancer. | DCIS is an early form of breast cancer and accounts for about 12% of all breast cancers. Breast cancer starts when cells in the breast begin to divide and grow in an abnormal way. Breasts are made up of lobules (milk-producing glands) and ducts (tubes that carry milk to the nipple), which are surrounded by glandular, fibrous and fatty tissue. When cancer cells have developed within the ducts of the breast but remain within the ducts (‘in situ’), it is called DCIS. The cancer cells have not yet developed the ability to spread outside these ducts into the surrounding breast tissue or to other parts of the body. As a result of being confined to the ducts, DCIS has a very good prognosis (outlook). | There is no way of knowing if/when DCIS will become invasive. Treatment removes DCIS to reduce the chance of it becoming invasive. If DCIS is left untreated, the cancer cells may develop the ability to spread outside the ducts, into the surrounding breast tissue. This is known as invasive breast cancer. Invasive cancer has the potential to also spread to other parts of the body | Some people find it helpful to discuss their feelings and concerns with their breast care nurse or specialist. If you’d like to talk through your feelings and concerns in more depth over a period of time, a counsellor or psychologist may be more appropriate. Your breast care nurse, specialist or GP (General Practitioner) can arrange this. Although DCIS is an early form of breast cancer with a very good prognosis, people understandably may feel very anxious and frightened by the diagnosis. People can often struggle to come to terms with being offered treatments such as a mastectomy, at the same time as being told their DCIS may never do them any harm. Some people are reluctant to say they’re anxious about a diagnosis of DCIS because they worry others will see it as less important than other types of breast cancer, or that they shouldn’t complain because they’re not having chemotherapy. Because of this they might feel less able to ask for support. But there are people who can support you so don’t be afraid to ask for help if you need it. By letting other people know how you feel, particularly your family and friends, they can be more supportive. | .....Some studies have found taking hormone therapy after surgery reduces the risk of DCIS coming back (recurrence) and the risk of invasive breast cancer developing, but the women taking it did not live longer than those who didn’t take it. | DCIS has a very good prognosis (outlook) | NR | Diagnosis DCIS is diagnosed using a range of tests, which may include one or more of the following: • breast examination • mammogram (breast x-ray) • ultrasound scan (uses high frequency sound waves to produce an image) • biopsy (removal of tissue to be looked at under a microscope) Surgery: Surgery is nearly always the first treatment for DCIS. This may be breast-conserving surgery or a mastectomy. Breast-conserving surgery, also known as a wide local excision or lumpectomy, is the removal of the DCIS with a margin (border) of normal breast tissue around it. A mastectomy is the removal of all of the breast tissue including the nipple area. Localisation: As most cases of DCIS can’t be felt, a procedure called localisation is often used on the day before or the day of breast-conserving surgery. Breast Reconstruction: If you’re having a mastectomy you’ll usually be able to have breast reconstruction. Lymph node removal: Most people with DCIS won’t have surgery to remove the lymph nodes. Adjuvant treatments: After surgery, you may need other treatments. These are called adjuvant treatments and can include radiotherapy and, in some cases, hormone therapy. Radiotherapy Radiotherapy uses high energy x-rays to destroy cancer cells. Hormone (endocrine) therapy A number of hormone therapies work in different ways to block the effect of oestrogen on cancer cells. | Four ways to get support: We hope this information was helpful, but if you have questions, want to talk to someone or read more about breast cancer, here’s how you can. Speak to our nurses or trained experts. Call our free Helpline on 0808 800 6000 (Monday to Friday 9am–4pm and Saturday 9am–1pm). The Helpline can also put you in touch with someone who knows what it’s like to have breast cancer. Chat to other women who understand what you’re going through in our friendly community, for support day and night. Look around, share, ask a question or support others at forum.breastcancercare.org.uk. Find trusted information you might need to understand your situation and take control of your diagnosis or order information booklets at breastcancercare.org.uk. See what support we have in your local area. We’ll give you the chance to find out more about treatments and side effects as well as meet other people like you. Visit breastcancercare.org.uk/in-your-area. | NR |
| Cancer Council Western Australia | Health professionals you may see: GP - assists with treatment descisions and works in partnership with your specialists in providing ongoing care, Breast Physician - diaganoses breast cancer and cooridinates treatment for breast cancer in some clinics, Breast Surgeon - specialises in surgery and performs biopsies; some breast surgeons also perform breast reconstruction and plastic surgery procedures, Oncoplastic Breast Surgeon - specialises in using plastic surgery techniques to reconstruct breast tissue after surgery, reconstructive (plastic) surgeon* - performs breast reconstruction for women who have had a mastectomy, anaesthetist - administers anaesthetic before surgery and monitors you during the operation, pathologist - examines cells and tissue samples that are removed from the breast to work out the type and extent of the cancer, breast care nurse - provides information, support and referral to women affected by breast cancer during and after treatment, nurse- administers drugs and provides care, information and support throughout treatment, radiologist - analyses x-rays, mammograms, ultrasounds and other scans, radiation oncologist - treats cancer by prescribing and coordinating a course of radiation therapy, radiation therapist - plans and delivers radiation therapy, medical oncologist - treats cancer with drug therapies such as chemotherapy, hormone therapy, targeted therapy and immunotherapy, lymphoedema practitioner - educates people about lymphoedema prevention and management, and provides treatment if lymphoedema occurs; often a physiotherapist, dietitian - recommends an eating plan to follow during treatment and recovery, exercise physiologist - prescribes exercise to help people with medical conditions improve their overall health, fitness, strength and energy levels, physiotherapist, occupational therapist - assist with physical and practical problems, including restoring movement and mobility after treatment, and recommending aids and equipment, social worker - links you to support services and helps you with emotional, practical or financial issues, genetic counsellor- provides advice for people with a strong family history of breast cancer or with a genetic condition linked to breast cancer, psychiatrist*, psychologist, counsellor - help you manage your emotional response to diagnosis and treatment | non-invasive breast cancer, abnormal cells | Ductal carcinoma in situ (DCIS) – abnormal cells in the ducts of the breast that may increase the risk of developing invasive breast cancer. | Remove the cancer and reduce the risk of it spreading or coming back | NR | DCIS is considered non-invasive breast cancer and usually develops into invasive breast cancer over time… What if the cancer returns? In the vast majority of cases, early breast cancer will not come back (recur) after treatment. Although the risk is higher with locally advanced breast cancer, most people will not experience a recurrence. However, it is possible for breast cancer to come back in the treated breast or in other parts of the body after treatment for early or locally advanced breast cancer. This is why it is important to have regular check-ups. | DCIS usually develops into invasive cancer over time. In most cases early cancer will not come back after treatment. It is possible for invasive cancer to come back in the same breast or other parts of the body after treatment for early cancer | NR | Diagnosis not specific to DCIS: Mammogram: A mammogram is a low-dose x-ray of the breast tissue. This x-ray can check any lumps or other changes found by the physical examination. Tomosynthesis – Also known as three-dimensional mammography or digital breast tomosynthesis (DBT), tomosynthesis takes x-rays of the breast from different angles and uses a computer to combine them into a three-dimensional image. Ultrasound: An ultrasound is a painless scan that uses soundwaves to create a picture of your breast. MRI: A magnetic resonance imaging (MRI) scan uses a large magnet and radio waves to create pictures of the breast tissue on a computer. Biopsy: During a biopsy, a small sample of cells or tissue is removed from your breast. Core biopsy, Vacuum Assisted Stereotactic Core Biopsy, Fine needle aspiration, Surgical Biopsy. In most cases, DCIS is treated in the same way as early invasive breast cancer. Surgery for early breast cancer will be either breast-conserving surgery, where part of the breast is removed, or a mastectomy, where the whole breast is removed. Nipple-sparing mastectomy with implant reconstruction: Ex. Right breast removed with a nipple-sparing mastectomy, followed by reconstruction using an implant. If it is not possible to keep the nipple, there is the option of having a nipple reconstruction later. Mastectomy with a flap reconstruction: Ex. Right breast removed, followed by reconstruction using tissue from the back. A reconstruction can also be done with tissue from the abdomen, buttock or thigh. Some women choose to have a nipple reconstruction later. Breast reconstruction: A breast reconstruction is a type of surgery in which a breast shape is created using a silicone implant, tissue from another part of your body, or a combination of both. Sentinel node biopsy – When breast cancer first spreads beyond the breast, it is likely to go to particular lymph nodes in the armpit or sometimes near the breastbone (sternum). Radiation therapy - Also known as radiotherapy, radiation therapy is the use of targeted radiation to kill or damage cancer cells so they cannot grow, multiply or spread. Hormone therapy - The aim of hormone therapy is to slow or stop the growth of hormone receptor positive cancer cells (tamoxifen, aromatase inhibitors, ovarian treatments) | NR | NR |
| Dr. Susan Love Research Foundation | NR | non-invasive, pre-cancer, abnormal cells | DCIS is a noninvasive precancer. It is not life threatening. If you have DCIS, it means that you have abnormal cells in the lining of a duct | No way of knowing which DCIS will become invasive or not. Treatment removes and prevents precancer from becoming invasive. | NR | While virtually all invasive cancer begins as DCIS, not all DCIS will go on to become an invasive cancer. An invasive cancer is one that has the potential to metastasize (spread)...Radiation will not increase your chances of survival (since DCIS is not life threatening) but it will reduce your risk of having the DCIS return as DCIS or invasive breast cancer....However, if the DCIS is ER-positive you will need to consider whether you want to take tamoxifen for five years to reduce your risk of a recurrence. | DCIS is not life-threatening | NR | DCIS appears as microcalcifications on a mammogram. When these microcalcifications are seen, it is recommended that a woman have a core biopsy or a wire localization biopsy. If the diagnosis is DCIS, make sure your pathology report includes information about the grade, presence of necrosis, margin, and estrogen receptor. This information is needed to determine how to treat the DCIS. How is DCIS treated? The options for treating DCIS are: lumpectomy, lumpectomy and radiation, a combination of those with tamoxifen, or mastectomy. Surgery: Most women undergo breast conservation surgery, a lumpectomy. However, if the DCIS is throughout the breast, a mastectomy will probably be necessary. Radiation: It is recommended that most women with DCIS receive radiation following a lumpectomy. Radiation will not increase your chances of survival (since DCIS is not life threatening) but it will reduce your risk of having the DCIS return as DCIS or invasive breast cancer. Hormone Therapy: Since DCIS is not capable of spreading, there is no reason to use chemotherapy. However, if the DCIS is ER-positive you will need to consider whether you want to take tamoxifen for five years to reduce your risk of a recurrence. The decision to take tamoxifen for DCIS is a difficult one for many women, as the benefits from taking it are small and have to be weighed against the risks associated with the drug as well as any side effects you may experience. If you decide to have genetic testing and if you are found to carry a BRCA genetic mutation your doctor may suggest that you consider a bilateral prophylactic mastectomy (removal of both breasts). This will reduce the chance of getting breast cancer by about 95 percent. | NR | NR |
| National Comprehensive Cancer Center | Your surgeon will make a large cut into your breast large enough to remove the cancer….Your doctors may suggest other options based on your health and other factors…Pathologist: A doctor who's an expert in testing cells and tissue to find disease. To rebuild a nipple a plastic surgeon can use surrounding tissues. | non-invasive breast cancer, stage 0 | DCIS (ductal carcinoma in situ) is a breast cancer that is confined to the ducts. It is a form of non invasive breast cancer. Cancer Grade: There are 3 grades of DCIS. Grade I looks the most like normal cells. It is the least likely to spread. Grade II also grows slowly. Grade III looks the least like normal cells. It is the most likely to spread. Grade III is often linked with comedo necrosis. | Prevent DCIS from invading stroma. | NR | Cancer cells can grow into surrounding tissues. If not treated, the primary tumor can grow through a duct or lobule into the stroma. Risk-reduction treatment may help prevent a second breast cancer. There are three main ways to reduce your risk. Talk with your doctor about which methods are right for you. Lifestyle changes: Changes in your lifestyle may reduce your chance of a second breast cancer. Eating a more healthy diet may help. You might need to exercise more. You might need to achieve a healthy body weight. Ask your doctor for a lifestyle plan that is right for you. | Not all DCIS will become invasive cancer. DCIS is not-life threatening so radiation will not increase your chances of survival but it will reduce the risk of having the DCIS return as DCIS or invasive cancer | NR | Treatment Planning: You doctor willask about any health problems and treatment during your lifetime….Physical Exam: A physcial exam is a study of your body. It is done to look for signs of disease…Clinical Breast Exam: Your doctor will look closely at and touch your bare breasts. The area around your breast will be viewed and touched...Imaging: Imaging tests make pictures of the insides of your body. They are used to find cancer...Diagnostic Bilateral Mammogram: A mammogram is a picture of the insides of your breast. The pictures are made using x-rays...Breast MRI: Breast MRI is not often used for DCIS. Your doctor may order it if the mammogram is unclear. Lab Tests: A pathologist is a doctor who`s an expert in testing cells to find disease. He or she will test the tissue that was removed from your body. Estrogen recpetor test: Estrogern is a hormone. maong some women, estrogen really helps the cancer cells grow....IHC (immunohistochemistry) is a lab test that detects hormone receptors...Pathology Report: All lab results are included in a pathology report....Genetic Counseling: Your disease or family history may suggest you have hereditary breast cancer. In this case your doctor will refer you for genetic counseling. Cancer Treatment: Lumpectomy + Radiation Therapy: A lumpectomy followed by radiation therapy is called breast-conserving therapy...It is an option for many but not all women with DCIS...A lumpectomy is a surgery that removes the tumour whiloe sparring healthy tissue...Some Normal-looking tissue around the tumours edge is also removed. This tissue is called a surgical margin.... Radiation therapy is given when the surgical margin is cancer free. It treats cancer cells that may remain in the breast after lumpectomy...Total Masectomy +/- sentinel lymph node biopsy: A total masectomy is a surgery that removes the whole breast.Operation is also called a simple masectomy. An SLNB (sentinel lymph node biopsy) finds and removes 2 or 3 of these nodes (first nodes to which lymph travels after leaving the breast. Lumpectomy Only, Clincal Trial: A clinical trial is a type of research the studies a test or treatment in people. Risk Reduction Treatment: Risk reduction treatment may help prevent a second breast cancer. Lifestyle changes: Changes in your lifestyle may reduce your chance of a second breast cancer...Endocrine therapy: Endocrine therapy includes treatments that stop cancer growth caused by hromones. It is sometimes called hormone therapy. Not the same as hormone replacement therapy. Endocrine therapy includes treatments that stop cancer growth caused by hormones. It is sometimes called hormone therapy. It is not the same as hormone replacement therapy. Talk to your doctor about starting endocrine therapy. It may help prevent a second breast cancer if you had estrogen receptor–positive cancer. It is unknown how well endocrine therapy works if you had estrogen receptor–negative cancer. Your doctor may advise taking endocrine therapy based on other factors, such as family history. Surgery (thrird method of risk reduction) ex. Bilateral salpingo-oophorectomy: This surgery removes both ovaries and fallopian tubes. | Follow-up care: Follow up care is important for your long term health. It is started after treatment ends. There must be no signs or symptoms of cancer. Medical History and physical exam: An update of your health history and physcial exam are part of follow-up care. Both should be done every 6 to 12 months for 5 years. After 5 years of normal results, these tests are needed once a year. Mammogram: A mammogram should be done every 12 months. The first one may be received as soon as 6 months after a breast conserving treatment. Mammograms arent needed if you had both breasts removed to reduce your cancer risk. After surgery some women choose to have breast reconstruction: Volume displacement: If you will have a lumpectomy your breast can be reshaped. This procedure is called volume displacement. Implants and flaps: breasts can be fully reconstructed with implants and flaps. All methods are generally safe, but as with any surgery there are risks. Implants: Breast Implants are small bag filled with salt water, silicine gel or both. They are places under the breast skin and muscle....Flaps: Breasts can be remade using tissue from your body, known as "flaps". Flaps are taken from the belly area, butt or from under the shoulder blade. Implants and flaps: Some breasts are reconstructed eith both implants and flaps. This method my give the recontructed breast more volume to match the other breast. Nipple Replacement: Like your breast, you can have your nipple remade. To re build a nipple, a plastic surgeon can use surround tissues. | Areas for Notes Included, NCCN Mamber Institutions List for Additonal Resource Info: ex. The Sidney Kimmel Comprehensive Cancer Centre at Johns Hopkins *Baltimore, Maryland 410.955.8964. hopkinskimmelcancercentre.org.* Questions to ask: You may meet with experts from different fields of medicine. Strive to have helpful talks with each person. Prepare questions before your visit and ask questions if the person isn’t clear. You can also record your talks and get copies of your medical records.  ex. Whats my diagnosis and prognosis? 1. Where did the cancer start? In what type of Cell? Is this cancer common? \| What are my options? 1. What will happen if I do nothing? \| What does each option require of me? 1. Will I have to go to the hospital or elsewhere? How often? How long is each visit? \| What is youre experience? 1. Are you board certified? |
| National Health Service | General Practicioner, Doctors (general term used), surgeon, specialist nurse, councellor, psychotherapist | non-invasive breast cancer, cancer, carcinoma in situ, Stage 0. | Non-invasive breast cancer is also known as cancer or carcinoma in situ. This cancer is found in the ducts of the breast and hasn't developed the ability to spread outside the breast. This form of cancer rarely shows as a lump in the breast that can be felt, and is usually found on a mammogram. The most common type of non-invasive cancer is ductal carcinoma in situ (DCIS). | NR | NR | By removing as much breast tissue as possible, a mastectomy can reduce your risk of breast cancer by up to 90%. (No specific information regarding recurrence or progression for DCIS) | NR | By removing as much breast tissue as possible, a mastectomy can reduce your risk of breast cancer by up to 90%. (NOT DCIS SPECIFIC regarding recurrence or progression) | Diagnosis: Mammogram and breast ultrasound: If you have symptoms and have been referred by your GP, you'll have a mammogram to produce an X-ray of your breasts. You may also need an ultrasound scan. Biopsy: A biopsy is where a sample of tissue cells is taken from your breast and tested to see if it's cancerous. Different methods of carrying out a biopsy are discussed: Needle aspiration may be used to test a sample of your breast cells for cancer or to drain a benign cyst (a small fluid-filled lump). Needle biopsy is the most common type of biopsy. Your doctor may suggest that you have a guided needle biopsy (usually guided by ultrasound or X-ray, but sometimes MRI is used) to obtain a more precise and reliable diagnosis of cancer and to distinguish it from any non-invasive change, particularly ductal carcinoma in situ (DCIS). Vacuum-assisted biopsy, also known as mammotome biopsy, is another type of biopsy. Further tests for breast cancer: Scans and X-rays Computerised tomography (CT) scans, or chest X-ray and liver ultrasound scans, may be needed to check whether the cancer has spread to your lungs or liver. During a hormone receptor test, a sample of cancer cells will be taken from your breast and tested to see if they respond to either oestrogen or progesterone. Surgery:There are two main types of breast cancer surgery. They are: surgery to remove the cancerous lump (tumour), known as breast-conserving surgery, surgery to remove the whole breast, which is called a mastectomy. In many cases, a mastectomy can be followed by reconstructive surgery to try to recreate a bulge to replace the breast that was removed. Studies have shown that breast-conserving surgery followed by radiotherapy is as successful as total mastectomy at treating early-stage breast cancer. Breast-conserving surgery: Breast-conserving surgery ranges from a lumpectomy or wide local excision, where just the tumour and a little surrounding breast tissue is removed, to a partial mastectomy or quadrantectomy, where up to a quarter of the breast is removed. Mastectomy: A mastectomy is the removal of all the breast tissue, including the nipple. Reconstruction: Breast reconstruction is surgery to make a new breast shape that looks as much as possible like your other breast. Lymph node surgery: To find out if the cancer has spread, a procedure called a sentinel lymph node biopsy may be carried out. Radiotherapy: Radiotherapy uses controlled doses of radiation to kill cancer cells. It's usually given after surgery and chemotherapy to kill any remaining cancer cells. Chemotherapy: Chemotherapy involves using anti-cancer (cytotoxic) medication to kill the cancer cells. Hormone treatment: Some breast cancers are stimulated to grow by the hormones oestrogen or progesterone, which are found naturally in your body (Tamoxifen, Aromatase Inihibitors, Ovarian Ablation or Suppression). Biological therapy (targeted therapy): Some breast cancers are stimulated to grow by a protein called human epidermal growth factor receptor 2 (HER2) (Trastuzumab), Clinical trials: A great deal of progress has been made in breast cancer treatment, and more women now live longer and have fewer side effects from treatment. Psycological Help: Dealing with cancer can be a huge challenge, for both patients and their families. It can cause emotional and practical difficulties. Complementary Therapy: Complementary therapies are holistic therapies that can promote physical and emotional wellbeing. | (General Information for Breast Cancer - DCIS is not differentiated) Living with breast cancer: Recovery and follow-up: Recovery: Most women with breast cancer have an operation as part of their treatment. Getting back to normal after surgery can take some time. It's important to take things slowly and give yourself time to recover. Follow-up: After your treatment has finished, you'll be invited for regular check-ups, usually every three months for the first year. If you've had early breast cancer, your healthcare team will agree a care plan with you after your treatment has finished. Long-term complications: Although it's rare, your treatment for breast cancer may cause new problems, such as: pain and stiffness in your arms and shoulders may occur after surgery, and the skin in these areas may be tight, lymphoedema. Your body and breasts after treatment: Dealing with changes to your body: A diagnosis of breast cancer may change how you think about your body. Early menopause: Although most cases of breast cancer occur in women over 50 who gave experienced the menopause, some younger women have to cope with an early menopause brought on by cancer treatment. Prosthesis: A breast prosthesis is an artificial breast, which can be worn inside your bra to replace the breast that's been removed. Relationships and sex: Relationships with friends and family. It's not always easy to talk about cancer, either for you or your family and friends. You may sense that some people feel awkward around you or avoid you. Breast cancer treatment can affect your sex life. Money and financial support: If you have to reduce or stop work because of your cancer, you may find it difficult to cope financially. Talk to other people: Your GP or nurse may be able to answer any questions you have about your cancer or treatment. You may find it helpful to talk to a trained counsellor or psychologist, or to someone at a specialist helpline. Your GP surgery will have information on these. Prevention: Diet and Exercise: Regular exercise and a healthy, balanced diet are recommended for all women because they can help prevent many conditions, including heart disease, diabetes and many forms of cancer. Breastfeeding: Studies have shown that women who breastfeed are statistically less likely to develop breast cancer than those who don't. | Sections with Additonal Links to External Reosurces (Not solely based on DCIS) : ex. Want to know more? Breast Cancer Care: Checking your breasts Breast Cancer Now: Signs and symptoms of breast cancer Want to know more? Breast Cancer Care: Referral to a breast clinic Breast Cancer Care: Your pathology report Macmillan: How breast cancer in women is diagnosed |
| Cancer Care Nova Scotia | NR | NR | NR | NR | Cancer is a physical disease but it also can affect how you think and feel. Your emotional health and well-being are very important as you go through your treatments. It is normal for you and your family to have many feelings at this time. You may feel anxious, frightened, worried, angry, or depressed. | NR | NR | NR | Why is radiation therapy used to treat breast cancer or DCIS? (Radiation Treatment Only) Breast cancer or DCIS may be treated with a combination of treatments such as surgery, radiation therapy, chemotherapy and hormone therapy. Radiation therapy uses radiation from high-energy X-ray machines to kill cancer cells. Radiation is used to lower the chance of the cancer returning in the breast, chest wall (and lymph node areas). | Common side effects include (radiation therapy): • Fatigue (Tiredness) › Fatigue caused by radiation therapy is usually mild. You may be more tired after a usual activity or may need to rest more than usual. You will be given an information sheet with suggestions to help you cope with the fatigue. • Skin reaction › Your skin in the treatment area may become dry and itchy, pink or even red. Some dark skinned patients may have increased darkening of the skin. › There may be small areas of dry or sometimes wet peeling of the skin. For those women who have not had a full mastectomy, there may be areas of tenderness, especially at the upper inner part of the breast, the nipple, or the crease underneath the breast. | Questions you may want to ask your Health Care Team • What will be the timing of my chemotherapy and radiation treatments? • Do I have to do anything to prepare for my radiation markings or treatment? • Can I bathe or wash? • What should I expect to happen during my treatments? • How will I cope with the side effects? • How will I know if the treatments are working? • What happens when the treatments end? • Do I need to come back for a checkup? • Will I have any long-term side effects from my treatments? • Who can I talk to if I have questions about supportive care; for example, medication costs, insurance, home care, transportation, emotional concerns, or any other questions? • Is there a research study for my cancer type that might be appropriate for me? (or that I could be involved in?) |
| Susan G. Komen | NR | non-invasive, abnormal cells, intra-ductal carcinoma, pre-invasive, pre-cancerous | Ductal carcinoma in situ (DCIS) is a non-invasive breast cancer that occurs in the milk ducts. The ducts carry milk from the lobules (where its made) to the nipple during breast feeding. DCIS is called "in situ" (which means "in place") because the abnormal cells are contained within the milk ducts and have not spread to nearby breast tissue. DCIS is also called intraductal (within the milk ducts) carcinoma. You may hear the terms "pre-invasive" or "pre-cancerous" to describe it. | No way of knowing which cases of DCIS will progress into invasive cancer. DCIS is treated to prevent invasive breast cancer development. | emotional health and well-being are very important as you go through your | Risk of developing invasive breast cancer after DCIS: Although prognosis is excellent after treatment for DCIS, theres still a small chance DCIS could return or invasive breast cancer could develop. | Prognosis is excellent after treatment for DCIS, but small chance of reccurance of DCIS or development of invasive breast cancer. | NR | Treatment for DCIS: Treatment for DCIS usually involves surgery, with or without radiation therapy. Some people may take hormone therapy. Be sure to discuss your treatment options with your doctor. Surgery: The first step in treating DCIS is surgery to remove the abnormal tissue in the breast. Depending on how far the DCIS has spread within the milk ducts, surgery can be lumpectomy or mastectomy. Lumpectomy: If there is little spread of DCIS within the milk ducts, a lumpectomy (also known as breast conserving surgery) can be done. The surgeon removes only the abnormal tissue, but the rest of the breast is left intact. In most cases, lymph nodes are not removed. It is usually day surgery (you do not need to stay overnight in the hospital). Mastectomy: If DCIS affects a large part of the breast, you will need a total (simple) mastectomy. The surgeon removes the entire breast, but no other tissue or lymph nodes. This treatment requires a short hospital stay. Breast reconstruction (surgery to recreate the breast) may be done at the time of the mastectomy or later. Radiation therapy: Radiation therapy uses high-energy X-rays to kill cancer cells. Lumpectomy for DCIS is usually followed by radiation to lower the risk of invasive breast cancer and DCIS recurrence (returning). Some women with smaller, lower grade DCIS and clean surgical margins, may be able to have lumpectomy without radiation therapy. Hormone therapy: Hormone therapy isn’t recommended for women who have a mastectomy for DCIS. (Prognosis is excellent without hormone therapy and so its benefit is likely very small.) | NR | Resources: Susan G. Komen® 1-877 GO KOMEN (1-877-465-6636) www.komen.org |
| Susan G. Komen | ...the surgeon removes only the abnormal tissue...A pathologist determines the hormone receptor status by testing the tissue removed during a biopsy. | non-invasive, intra-ductal carcinoma, pre-invasive, pre-cancerous | DCIS (ductal carcinoma in situ) is a non-invasive breast cancer. In DCIS, the abnormal cells are contained in the milk ducts (canals that carry milk from the lobules to the nipple openings during breastfeeding). It’s called “in situ” (which means "in place") because the cells have not left the milk ducts to invade nearby breast tissue. DCIS is also called intraductal (within the milk ducts) carcinoma. You may hear the terms “pre-invasive” or “pre-cancerous” to describe DCIS. | No way of knowing which cases of DCIS will progress to invasive breast cancer. DCIS is treated to prevent invasive breast cancer development. | treatments. It is normal for you and your family to have many feelings at this | Risk of developing invasive breast cancer after DCIS: After treatment for DCIS there's a small risk of: DCIS recurrence or Invasive breast cancer. These risks are higher with lumpectomy plus radiation therapy than with mastectomy [2]. However, overall survival is the same after either treatment [2]. With close follow-up, invasive breast cancer is usually caught early and can be treated successfully. | Prognosis for DCIS with treatment is ussualy excellent; after treatment small risk of DCIS reccurance or invasive breast cancer | NR | Prognosis: With treatment, prognosis for DCIS is usually excellent. Surgery: Surgery is the first step to treat DCIS. It removes the abnormal tissue from the breast. Depending on how far the DCIS has spread within the milk ducts, surgery can be mastectomy or lumpectomy. If DCIS has spread throughout the ducts, affecting a large part of the breast, a total (simple) mastectomy will be done. In a total mastectomy, the surgeon removes the entire breast and possibly some lymph nodes, but no other tissue. If there's little spread of DCIS within the ducts, a choice can be made between mastectomy or lumpectomy. With lumpectomy, the surgeon removes only the abnormal tissue, and the rest of the breast is left intact. Lymph nodes are not usually removed with lumpectomy for DCIS. In the U.S., most women with DCIS are treated with lumpectomy followed by radiation therapy [3]. Sentinel node biopsy and mastectomy for DCIS: A sentinel node biopsy is a procedure used to check whether or not invasive breast cancer has spread to the lymph nodes in the underarm area (axillary nodes). It removes 1-5 nodes. Having a sentinel node biopsy during a mastectomy helps some people with DCIS avoid an axillary dissection. Once a mastectomy has been done, a person can no longer have a sentinel node biopsy. If it turns out there’s also invasive breast cancer (in addition to DCIS) in the tissue removed during the mastectomy, the sentinel node biopsy will have already been done. If a sentinel node biopsy was not done and invasive breast cancer is found, an axillary dissection will be needed. An axillary dissection removes more axillary lymph nodes than a sentinel node biopsy and increases the risk of problems such as lymphedema. So, even though a sentinel node biopsy may not be needed with DCIS, most people who have a mastectomy for DCIS will also have a sentinel node biopsy done at the same time. Radiation therapy: After mastectomy: Radiation therapy is almost never given to women treated with mastectomy for DCIS. After lumpectomy: Lumpectomy for DCIS is usually followed by radiation therapy to lower the risk of [2-9]: DCIS recurrence Invasive breast cancer Hormone therapy: Hormone receptor status. A pathologist determines the hormone receptor status by testing the tissue removed during a biopsy. Hormone receptor-positive (estrogen receptor-positive/progesterone receptor-positive) DCIS tumors express (have a lot of) hormone receptors. Hormone receptor-negative (estrogen receptor-negative/progesterone receptor-negative) DCIS tumors do not express (have few or no) hormone receptors. Hormone receptor-positive DCIS may benefit from hormone therapy (tamoxifen or an aromatase inhibitor) [2,6,12]. | NR | Question Prompt List: Is ductal carcinoma in situ (DCIS) breast cancer? How does DCIS differ from invasive breast cancer? What are my treatment options? Which treatments do you recommend for me and why? What are my chances for DCIS recurrence? What about developing invasive breast cancer? How long do I have to make a decision about my treatment plan? Can I have a lumpectomy (breast conserving surgery)? If not, why not? If I have a lumpectomy plus radiation therapy now, and the breast cancer returns (DCIS recurrence or invasive breast cancer) in the future, will I need to have a mastectomy at that time? Will I need radiation therapy after my surgery? If I have radiation therapy, when will the radiation oncologist discuss my radiation treatment with me? If I have a mastectomy, will a sentinel node biopsy also be done? Is my DCIS estrogen receptor-positive or -negative? Will I need hormone therapy, such as tamoxifen or an aromatase inhibitor? Were my tumor margins negative (also called uninvolved, clean or clear)? If not, what more will be done? Tell me about breast reconstruction. If I decide I want reconstruction, when can I have it (at the same time as the mastectomy or at a later date)? What are the risks? What about prosthesis options? Who else should I see to discuss and plan for reconstruction or prosthesis? How often will I have check-ups and follow-up tests after treatment ends? Will a follow-up care plan be prepared for me? Which health care provider is in charge of my follow-up care? Is there a clinical trial enrolling people with DCIS? If so, how can I learn more? Will some of the tissue removed during surgery be saved? Where will it be stored? For how long? How can it be accessed in the future? |
| Ohio State University Comprehensive Cancer Center | NR | abnormal cells, non-invasive cancer, Stage 0 breast cancer, cancer cells | What is DCIS? Ductal Carcinoma in Situ, or DCIS, is a cluster of abnormal cells found in a milk duct in your breast. Milk ducts are tubes that carry milk from the lobules, where breastmilk is made, to your nipple during breastfeeding. This condition is called “non-invasive cancer” or a Stage 0 breast cancer. DCIS refers to cancer cells that are only in your milk ducts. The cancer cells have not spread outside your milk ducts or to any other area of your body. Most other breast cancers are found once they have spread to other breast tissue. | Tests cannot predict if DCIS will become invasive. DCIS is treated to get rid of all abnromal cells. | time. You may feel anxious, frightened, worried, angry, or depressed. | How to prevent breast cancer after DCIS diagnosis • It is important to check with your doctor if you should stop: ⊲ Birth control pills or shots (it is important to talk with your doctor about other birth control methods you can use) ⊲ Estrogen and progesterone (pills, patches, vaginal rings or creams) ⊲ Plant estrogen supplements (natural estrogen) • Try not to eat or drink a lot of soy products, such as soybeans, soy milk, and tofu. • Your doctor may order anti-hormone therapy pills for you to take after your surgery. This can help to protect your breast tissue and prevent breast cancer. | NR | NR | How is DCIS diagnosed? DCIS is often found during a routine mammogram. If your mammogram is abnormal, a breast biopsy is done. How is DCIS treated? You and your doctor will decide your treatment plan based on: • Your mammogram results • Your biopsy results • Your breast exam results • The location of the abnormal cells in your breast • The area or areas of DCIS in your breast • How much breast tissue is going to be removed. Surgery If your doctor recommends surgery you may have one of the following: • Lumpectomy – removal of the abnormal area and a rim of normal tissue (clear margin) around it. If abnormal cells are found in the clear margin, you may need more surgery. You may have radiation therapy after this surgery. Total mastectomy - removal of your whole breast, including your breast tissue, skin, and nipple. Your lymph nodes may also be removed and checked for abnormal cells. Your doctor may talk with you about reconstructive surgery to create a mound of tissue where your breast has been removed. | Close Follow-Up It is important to have routine checkups after your diagnosis. This includes a mammogram each year, monthly self-breast exams and a breast exam by your doctor every 3 to 6 months. It is important for you to do self-breast exams each month and tell your doctor or nurse about any changes you may have. | NR |
| The Pennine Acute Hospitals | Introduction to the Surgical Breast Team: Before your surgery the consultant surgeon who specialises in treating breast conditions will explain the operation to you. You are likely to meet other surgeons, Advanced Nurse Practitioners (ANP's),the radiology team and Clinical Nurse Specialists(CNS) who all work as part of the team. The team works closely together to ensure that you receive a quality service. In addition to considering the type of breast cancer you have, your doctors will also look at its other characteristics to help decide on the most appropriate treatment for you. | early form of breast cancer, pre-cancer, intra-ductal cancer, non-invasive cancer | Ductal carcinoma in situ (DCIS) is an early form of breast cancer, sometimes described as pre-cancer, intraductal or non-invasive cancer. The cancer cells are inside the milk ducts (in situ) and have not yet developed the ability to spread either outside the ducts into surrounding breast tissue or to other parts of the body. There are different grades of DCIS...With ductal carcinoma in situ (DCIS) the three grades are usually called low, intermediate and high instead of 1, 2 or 3. | Without treatment DCIS may become invasive cancer. Treatment aims to remove cancer before it is able to spread. | NR | It is thought that if DCIS is left untreated, the cells may become invasive cancer. DCIS may, if left untreated, develop the ability to spread outside of the ducts, known as invasive cancer. However in some cases DCIS will never develop further. Studies have shown that long-term survival is approximately the same for breast conserving surgery (sometimes followed by radiotherapy) as for a mastectomy. | In some cases DCIS will never develop further | NR | Treatment will usually include surgery (either breast-conserving surgery or a mastectomy with or without reconstruction) to remove the affected area. Breast-conserving surgery may be followed by radiotherapy. Once the tissue has been removed and examined, it may be that invasive cancer is found as well as non-invasive cancer. If this is the case it may alter your recommended treatment. Breast-conserving surgery: Usually referred to as wide local excision or lumpectomy. The cancer is removed with a margin of normal breast tissue. Mastectomy: This means removal of all the breast tissue including the nipple area. A simple mastectomy means that the entire breast is removed but the lymph nodes in the armpit and the muscles underneath the breast are not affected – although some lymph nodes may be removed with the breast tissue taken during surgery. Breast prosthesis If you have a mastectomy you may want to wear a prosthesis – a false breast form. | After your operation: As part of the enhanced recovery programme you will be expected to play an active role in your recovery as this will help you to feel well sooner. Dressings: ..If you have any concerns regarding your wound or dressings after your operation then please contact your CNS or the ward. Moving around after surgery: ...Being out of bed and in an upright position plus walking regularly helps to improve lung function and reduce the risk of a chest infection. Drains: After breast surgery you may have one or more surgical drains in place near to your wound. Nausea and sickness: The majority of patients do not experience problems with nausea or sickness. However, if you experience nausea thisis usually caused by the anaesthetic drugs and should soon settle. Complications of surgery: ...Undergoing surgery is not without risk. Being on the enhanced recovery programme will considerably reduce the risk of developing complications and reduce your length of stay. Infection: The nurses and doctors take great care to prevent infection when operating or attending to wounds and drains...Cardiovascular problems: This is a complication associated with any surgery and anaesthetic....Blood clots and Signs of a possible blood clot. What to expect after surgery: The following are all possible after-effects. Some people may experience some or all of these whilst others may experience none: Pain and discomfort: You are likely to have pain or discomfort after surgery...Bruising and swelling: Bruising is common after surgery but will disappear over time...Seroma, Wound infection, Haematoma, Change in sensation, Stiffened shoulder, Cording, Arm swelling (lymphoedema) Returning home after breast surgery: Although you should be feeling well when you are discharged you may find even simple tasks leave you feeling exhausted. Things you should do: *Get bed and get dressed everyday. *Accept offers of help, *Gentle exercise, *Eat/drink Normal, *Painkillers. Things you should not do: *Do not lift heavy items,*Do not drive, *Avoid Heavy household chores, *Do not return to work until meeting with consultant | If you have any problems or concerns within normal office hours (Monday-Friday 9.00am - 5.00pm) please contact the CNS offiEe on 0161 720 2558 (North Manchester General Hospital) or 0161 627 8459 (The Royal Oldham Hospital). Other useful contact numbers: Beechwood Cancer Care Centre: 0161 476 0384 Breast Cancer Care: 0800 800 6000 www.breastcancercare.org.uk Bury Cancer Support Centre: 07899 990260 Mondays, Tuesdays and Wednesdays 10:30am – 4pm Cancer Aid Network (Oldham): 01457 874927 Chaplaincy: • North Manchester - 0161 720 2990 • Oldham - 0161 627 8796 • Bury - 0161 778 3568 • Chaplaincy Co-ordinator - 0161 778 5259 Citizens Advice Bureau: Telephone: 03444 889 622 10.00 am - 4.00 pm - Monday to Friday ETC.... |
| University of Iowa Hospitals and Clinics | We have a multidisciplinary team approach to the treatment of breast cancer. Surgical. medical and radiation oncologists work together with pathologists, plastic and reconstructive surgeons, nurses, genetics counselors and pharmacists to develop an individualized treatment plan that is tailored to you and your specific type of breast cancer. (mention of who is involved in care without description) | non-invasive type of breast cancer, Stage 0 cancer | Ductal Carcinoma in Situ (DCIS) is a non-invasive type of breast cancer that occurs when abnormal cells are found solely within the milk ducts. The words “in situ” means that the cells have remained in place and have not spread outside of the ducts into surrounding breast tissue. DCIS is considered to be a non-invasive breast cancer, which is why it’s called a Stage 0 cancer. | NR | NR | NR | Radiation therapy minimizes the risk of cancer returning or spreading. | NR | How is it diagnosed?: If a suspicious area is found on a routine mammogram, the radiologist will recommend an additional mammogram—called a diagnostic mammogram—to take a closer look. A breast tissue biopsy also may be scheduled. Treatment Options: Treatment options depend upon how large an area has DCIS or if there are multiple areas. Recommendations also will take into account your family medical history of cancer.  Lumpectomy This is surgery to remove the tissue where DCIS is detected, as well as some surrounding healthy tissue. Lumpectomy without any additional treatment is designed for patients who have a small, clearly defined area of DCIS. Lumpectomy plus radiation therapy This option, which follows surgery with a determined number of external beam radiation treatments, is the most common treatment plan for DCIS. The radiation therapy minimizes the risk of the cancer returning or spreading. Lumpectomy plus tamoxifen In some cases, cancer doctors will recommend a lumpectomy followed by the drug tamoxifen. Tamoxifen blocks the hormone estrogen, which, in some cases, can promote tumor growth and the spreading of cancer cells. Your doctor will tell you if hormones are impacting your specific cancer. Mastectomy A mastectomy, where the surgeon removes the breast tissue, skin, areola and nipple, is recommended if DCIS is found in a large portion of the breast, or if there are multiple areas of abnormal cells. It’s also recommended if the area of DCIS does not have clearly defined margins (meaning abnormal cells are near the edges of the area and increase the risk of spreading beyond the ducts). Nipple-Sparing Mastectomy At University of Iowa Health Care, breast and plastic surgeons also offer nipple-sparing mastectomy. This procedure allows you to keep some of your breast skin, including the nipple and areola, after mastectomy. | NR | NR |
| American Society of Clinical Oncology | NR | non-invasive cancer | Cancer that has not spread past the ducts and is not invasive | NR | NR | NR | Additonal treatment with chemtherapy, raditation therapy, hormonal therapy or targetted therapy is given after suregry to reduce the risk of cancer returning. | NR | General Breast Cancer Info Given: DCIS Specific Info not specified. The biology and behavior of a breast cancer affect the treatment plan, and every person’s cancer is different. Doctors consider many factors when recommending a treatment plan, including the cancer’s stage; the tumor’s human epidermal growth factor receptor 2 (HER2) status and the hormone receptor status, which includes estrogen receptors (ER) and progesterone receptors (PR); the presence of known mutations (changes) in breast cancer genes; and the woman’s age, general health, and whether she has experienced menopause. For earlier stages of cancer, surgery to remove the tumor and nearby lymph nodes usually is the first treatment. Additional treatment with chemotherapy, radiation therapy, hormonal therapy, or targeted therapy is usually given after surgery to lower the risk of the cancer returning. These treatments may also be given before surgery to shrink the size of the tumor. The treatment of cancer that has spread or come back after treatment depends on many factors. It can include the therapies listed above used in a different combination or at a different pace. When making treatment decisions, women may also consider a clinical trial; talk with your doctor about all treatment options. The side effects of breast cancer treatment can be reduced or managed with a variety of medications and the help of your health care team. This is called palliative care and is an important part of the overall treatment plan. | NR | NR |
| BreastCancer.org | NR | non-invasive breast cancer | Ductal carcinoma in situ (DCIS) is non-invasive breast cancer. Ductal means that the cancer starts inside the milk ducts, carcinoma refers to any cancer that begins in the skin or other tissues (including breast tissue) that cover or line the internal organs, and in situ means "in its original place." DCIS is called "non-invasive" because it hasn’t spread beyond the milk duct into any normal surrounding breast tissue. There are three grades of DCIS: low or grade I; moderate or grade II; and high or grade III. The lower the grade, the more closely the cancer cells resemble normal breast cells and the more slowly they grow. Sometimes it's difficult to figure out where the cells are on in the range from normal to abnormal. If the cells are in between grades, they may be called "borderline." | NR | NR | DCIS isn’t life-threatening, but having DCIS can increase the risk of developing an invasive breast cancer later on. When you have had DCIS, you are at higher risk for the cancer coming back or for developing a new breast cancer than a person who has never had breast cancer before. Most recurrences happen within the 5 to 10 years after initial diagnosis. The chances of a recurrence are under 30%. | When you have had DCIS you are at a higher risk for cancer coming back or for developing new breast cancer, than a person who has not had breast cancer before. | Most recurrences happen within 5-10 years of initial diagnosis. The chances of a recurrence are under 30% | Diagnosis of DCIS: Physical examination of the breasts: Your doctor may be able to feel a small lump in the breast during a physical examination, although a noticeable lump is rare with DCIS….Mammography: DCIS is usually found by mammography. As old cancer cells die off and pile up, tiny specks of calcium (called "calcifications" or "microcalcifications") form within the broken-down cells. Biopsy: If you do have a suspicious mammogram, your doctor will probably want you to have a biopsy....Fine needle aspiration biopsy: A very small, hollow needle is inserted into the breast....Core needle biopsy: A larger needle is inserted to remove several bigger samples of tissue from the area that looks suspicious... If a needle biopsy is not able to remove cells or tissue, or it does not give definite results (inconclusive), a more involved biopsy may be necessary. These biopsies are more like regular surgery than needle biopsies: Incisional biopsy: Incisional biopsy removes a small piece of tissue for examination. Excisional biopsy: Excisional biopsy attempts to remove the entire suspicious lump of tissue from the breast. Treatment for DCIS: Lumpectomy followed by radiation therapy: Lumpectomy removes the entire area of DCIS as well as a margin of normal, healthy breast tissue around it. The whole area that contained cancer cells is removed, even when there's no lump present. Re-excision lumpectomy is a second surgery that may be necessary after lumpectomy to remove extra tissue in order to ensure that there is a clear margin of healthy tissue around the tumor. External radiation is given to the entire breast by a machine called a linear accelerator. Radiation treatment is usually given as daily treatments 5 days per week over 5 to 7 weeks. Internal partial-breast irradiation is a form of treatment in which radioactive materials such as seeds or pellets are temporarily placed in the breast. External partial-breast irradiation is a method of therapy that zeroes in on the area around where the cancer was. Mastectomy: Mastectomy, or removal of the breast, is recommended in some cases. In most DCIS cases requiring mastectomy, simple or total mastectomy (removal of breast tissue but no lymph nodes) is performed. Lumpectomy alone. Hormonal therapy after surgery: These treatments, which block or lower the amount of estrogen in the body, are typically used if the DCIS tests positive for hormone receptors. Hormonal therapy: People with DCIS have a slightly higher risk of developing another breast cancer in the future than people who have not had DCIS. Adding hormonal therapy to surgery and radiation for DCIS can reduce this risk if the tumor tests positive for hormone receptors (tamoxifen, aromatase inhibitors). Chemotherapy, a form of treatment that sends anti-cancer medications throughout the body, is generally not needed for DCIS. DCIS is non-invasive and remains within the breast duct, so there is no need to treat cancer cells that might have traveled to other areas of the body. The Oncotype DX DCIS test is a genomic test that can help you and your doctor make decisions about treatments after surgery for DCIS. | After surgery and radiation therapy, you and your doctor will work together to develop a plan for your follow-up care. If you had a mastectomy and are undergoing breast reconstruction, you will have a series of office visits to check on your healing. If you are taking tamoxifen or another form of hormonal therapy, this usually continues for a period of about 5 years, so your doctor will want to monitor you throughout that time. Although follow-up care plans can vary from person to person, your plan is at least likely to include: a checkup and physical exam by your doctor every 6 to 12 months for 5 years and then once a year after that and a mammogram every 12 months and possibly other screening methods depending on your doctor’s recommendations | NR |
| Breast Screen Aotearoa | NR | early form of breast cancer, cancerous but non-invasive | Ductal carcinoma in situ (DCIS) is a very early form of breast cancer that affects the breast ducts, which carry milk to the nipple. It is a growth that is cancerous but non-invasive. This means that it has not spread into the surrounding breast tissue and/or other areas of the body, but is contained within a breast duct. DCIS is often split into three groups: high grade, intermediate grade and low grade. The grading of DCIS (ie, high, intermediate or low) indicates how actively the abnormal cells are multiplying. Therefore, high-grade DCIS is when the cells are dividing more rapidly than low grade. High-grade DCIS is more likely to progress to high-grade aggressive invasive cancer. Intermediate grade represents DCIS that lies between low and high grade. Some low-grade DCIS either does not progress or takes many years to progress to invasive cancer. At present it is not known which low-grade DCIS is safe to leave in the breast, so treatment is almost always necessary. | Without treatment DCIS may spread becoming invasive and life threatening. No way of knowing which cases of DCIS will become invasive breast cancer. At present it is not known which low-grade DCIS is safe to leave in the breast, so treatment is almost always necessary. | NR | Recurrence after local excision (the return of cancer at the site of surgery) is relatively uncommon. Recurrence after mastectomy is rare. The recurrence rate is less than 1 percent per year. Hormone therapy (anti-oestrogen treatment) such as tamoxifen is sometimes recommended as an additional form of treatment to prevent recurrence or reduce the risk of a new cancer or DCIS in the other breast. | Not all cases of DCIS will become invasive cancer. High-grade DCIS is more likely to progress to high-grade aggressive invasive cancer. Intermediate grade represents DCIS that lies between low and high grade. Some low-grade DCIS either does not progress or takes many years to progress to invasive cancerRecurrence after local excision is relatively uncommon. Recurrence after masectomy is rare. Hormone therapy is sometimes recommended to prevent cancer recurrence/redcue risk of new cancer or DCIS. | Recurrence rate after local excision is less than 1 percent per year. | Most cases of ductal carcinoma in situ are detected by screening with mammography. This is done by core biopsy, which involves inserting a special biopsy needle into the affected area in the breast. Mammography or ultrasound is used to guide the needle into the correct spot and then several fi ne slivers of tissue are taken. Occasionally, the diagnosis cannot be made from needle samples. The tissue will then have to be obtained surgically. This is called an open biopsy. The affected area may need to be identifi ed and marked by inserting a small, very fi ne wire (called a hook wire) into the breast. Surgery: Your surgeon may recommend local excision or removal of the DCIS (breast-conserving surgery) along with some surrounding tissue. This is most commonly used for small areas of DCIS. Mammograms can sometimes underestimate the size and extent of the DCIS, particularly if it is low grade. This sometimes means that further surgery (another operation) needs to occur to remove all of the DCIS. • After breast-conserving surgery, radiation therapy may be recommended. • Mastectomy is recommended for women who have a large area of DCIS or several separate areas of DCIS within the breast. DCIS alone does not spread to the lymph nodes in the armpit (axillary nodes), so it is not usual to remove them. Lymph node surgery on the armpit may be advised in the following situations: • If you have a large area of DCIS, especially if this is high grade, you may have a sentinel node biopsy at the time of removal of the DCIS. • If invasive cancer is found after removal of the DCIS, your surgeon may then advise removal of lymph nodes from your armpit. • If you choose to have a mastectomy with immediate reconstruction, it is common to take some of the axillary nodes for testing. Hormone therapy (anti-oestrogen treatment) such as tamoxifen is sometimes recommended as an additional form of treatment to prevent recurrence or reduce the risk of a new cancer or DCIS in the other breast. | Follow-up treatment: All women treated for DCIS should have long-term, regular clinical examinations and annual mammograms to detect recurrence or a new cancer in the other breast. Most recurrences of DCIS are picked up by mammography. Free annual mammograms are available through your DHB on referral by a GP or hospital specialist. After fi ve years, if still in the eligible age range, you may have two-yearly mammograms withBreastScreen Aotearoa. | NR |
| Cancer Australia | NR | non-invasive breast cancer, abnormal cells | Ductal carcinoma in situ (DCIS) is the name for abnormal changes in the cells in the milk ducts of the breast. 'In situ' means 'in place'. DCIS is a non-invasive breast cancer. The abnormal cells are contained inside the milk ducts. | Unsure which cases of DCIS will become invasive breast cancer. Prevent possible development of invasive breast cancer. Without treatment, DCIS may become invasive. | NR | If DCIS is not treated it may develop into invasive breast cancer, which can spread outside the ducts into the breast tissue and then possibly to other parts of the body. We don’t know for certain how many women with DCIS would develop invasive breast cancer if they were not treated. Also, it is not possible to predict which women with DCIS will develop invasive breast cancer if they were not treated or how long after the diagnosis of DCIS an invasive breast cancer would develop. Some women with DCIS may never develop any problems if they are not treated. However, some women with DCIS may develop invasive breast cancer. | Not all cases of DCIS will become invasive cancer. Recurrence after local excision is relatively uncommon. Recurrence after masectomy is rare. Hormone therapy is sometimes recommended to prevent cancer recurrence/redcue risk of new cancer or DCIS. | NR | Diagnosis: Most cases of DCIS are found following routine screening with mammograms. DCIS often appears as small flecks of calcium (called microcalcifications) on a mammogram or ultrasound. Treatment Options: Surgery for DCIS usually involves breast conserving surgery. Sometimes a mastectomy may be recommended. Find out when a mastectomy may be recommended for DCIS. Breast reconstruction may be possible after a mastectomy for DCIS. For some women, the surgical biopsy to diagnose DCIS is the only surgery needed because all the DCIS is removed by the biopsy. Radiotherapy is usually recommended after breast conserving surgery for women with DCIS. Radiotherapy is not usually recommended after mastectomy for women with DCIS. Surgery to remove lymph nodes: Because DCIS cells are contained within the milk ducts and don’t spread into the breast tissue, most women with DCIS don’t need to have lymph nodes removed from the armpit. Rarely, if the DCIS covers a large area of the breast or if the DCIS is high grade, removal of some lymph nodes may be recommended. Hormonal therapies are drugs that change the level of female hormones in the body or stop cells from being affected by hormones. There are several different types of hormonal therapy. Hormonal therapies might be effective in women with DCIS. However, the long-term benefits and side effects of hormonal therapies for women with DCIS are not yet known. Clinical trials are continuing to test hormonal therapies for treating DCIS. | NR | NR |
| Cancer Research UK | NR | cancer cells | DCIS means that some cells in the lining of the ducts of the breast tissue have started to turn into cancer cells. These cells are all contained inside the ducts. They have not started to spread into the surrounding breast tissue. The grade of DCIS tells you how much the cells look like normal breast cells. It gives your doctor an idea of how the DCIS might behave and which treatment you need. DCIS grade is divided into: low grade (more slowly growing), intermediate grade, and high grade (more quickly growing) | NR | NR | How likely is DCIS to come back: The chance of the DCIS coming back depends on various factors. But after mastectomy DCIS almost never comes back. In women who have just the area of DCIS removed the chance of it coming back is a bit higher. But it depends on the grade and type of DCIS. Your doctor can give you more information about the chance of the DCIS coming back in your case. | Those with DCIS may develop breast cancer if not treated. Uncertain how many women would develop invasive breast cancer without treatment. Women with DCIS may or may not develop invasive breast cancer in the future. | NR | DCIS is diagnosed more often now than in the past. It is often picked up in women when they have mammograms as part of the UK breast screening programme... Treatment for DCIS Surgery is the main treatment for DCIS.  You might have surgery to remove:  an area of the breast (wide local excision) the whole breast (mastectomy)...Removal of part of the breast Many women have surgery to remove the area of DCIS and a border of healthy tissue around it. This is called a wide local excision (WLE) or breast conserving surgery, or sometimes a lumpectomy...you might have radiotherapy to the rest of the breast tissue if the DCIS cells look very abnormal (high grade). The radiotherapy treatment aims to kill off any abnormal cells that might still be in the breast tissue....Removal of the whole breast You might have a mastectomy if:  the area of the DCIS is large there are several areas of DCIS you have small breasts and too much of the breast is affected by DCIS to make wide local excision possible...If you want to, you can choose to have a new breast made (breast reconstruction) at the time of the mastectomy, or some time afterwards....Hormone therapy Doctors are researching the role of tamoxifen and other hormone therapies as a treatment for DCIS. | Follow up After treatment you usually have regular check ups. At the check ups your doctor or a breast care nurse will examine you and ask about your general health. This is your chance to ask questions and to tell them if anything is worrying you.  How often you have check ups depends on your individual situation but they might go on for at least 5 years. This might include yearly mammograms.   It’s important to remember that you can contact your doctor or nurse between appointments if you are worried about a symptom or have questions. You don't have to wait for your next appointment. You can also speak to your GP.  In some hospitals you don't have regular appointments after treatment. But if you have new symptoms or are worried about anything you can phone your doctor or breast care nurse or make an appointment to see them.   UK guidelines say that everyone who has had treatment for early breast cancer should have a copy of a written care plan. The care plan has information about tests you will have, and signs and symptoms to look out for. It will also include contact details for specialist staff, such as your breast care nurse. | NR |
| Cancer Treatment Centers of America | NR | cancerous cells | Ductal carcinoma in situ (DCIS) is characterized by cancerous cells that are confined to the lining of the milk ducts and have not spread through the duct walls into surrounding breast tissue. If DCIS lesions are left untreated, over time cancer cells may break through the duct and spread to nearby tissue, becoming an invasive breast cancer. DCIS is divided into several subtypes, mainly according to the appearance of the tumor. These subtypes include micropapillary, papillary, solid, cribriform and comedo. | If DCIS lesions are left untreated, over time cancer cells may break through the duct and spread to nearby tissue, becoming an invasive breast cancer. | NR | Women with DCIS are typically at higher risk for seeing their cancer return after treatment, although the chance of a recurrence is less than 30 percent. Most recurrences occur within five to 10 years after the initial diagnosis, and may be invasive or noninvasive. DCIS also carries a heightened risk for developing a new breast cancer in the other breast. A recurrence of DCIS will require additional treatment. | After mastectomy DCIS almost never comes back. In women who have just the area of DCIS removed the chance of it coming back is a bit higher. Recurrence depends on the grade and type of DCIS. | Ductal carcinoma treatment options: The type of therapy selected may affect the likelihood of recurrence. Treating ductal carcinoma in situ with a lumpectomy (breast-conserving surgery) without radiation therapy carries a 25 – 35 percent chance of recurrence. Adding radiation therapy to the treatment decreases this risk to approximately 15 percent. Currently, the long-term survival rate for women with DCIS is nearly 100 percent. |  | NR | NR |
| Health Talk.org | NR | cancer cells, early form of breast cancer, pre-invasive cancer, non-invasive cancer, intra-ductal cancer | DCIS stands for ductal carcinoma in situ. This means the cancer cells are inside the milk ducts or ‘in situ’ (in place) and have not developed the ability to spread either within or outside the breast. DCIS is an early form of breast cancer and may be described as a pre-invasive, non-invasive or intraductal cancer. It may affect just one area of the breast, but can be more widespread and affect different areas at the same time. There are three grades of DCIS: low, intermediate, and high. The grade relates to how the cells look under the microscope, and gives an idea of how quickly the cells may develop into an invasive cancer (or how likely it is that the DCIS will come back after surgery). Low-grade DCIS has the lowest risk of developing into an invasive cancer, and high-grade carries the greatest risk. | Prevent DCIS development into invasive breast cancer. | Mastectomy for DCIS: physical and emotional recovery: At home, most women said they were careful not to lift anything heavy or over-exert themselves physically, including with housework. Some said they had a bit of pain or discomfort around their wound and under their arm to begin with, and took painkillers to ease it. Many said they had support from family until they felt able to do everything themselves again. A few said they had a visit from a district nurse. How DCIS affects families: Some women with DCIS thought that the diagnosis was possibly more shocking and disturbing for their close family and friends than it was for themselves. Support from family, friends and health professionals: Women often said that they had been very pleasantly surprised to discover how kind and helpful their families, partner, children, friends and work colleagues could be. Practical as well as emotional support was greatly appreciated' a husband might massage an aching back; a friend or family member might accompany the woman to an appointment, cook a meal or encourage her to keep up her social life; a friend might collect children from school or look after small children so that the woman and her partner could attend appointments together. One woman expressed how grateful she was that her sister arranged to be there when she came round from surgery. Another said that her family were wonderful at keeping her spirits up and could even joke about it. | The grade relates to how the cells look under the microscope, and gives an idea of how quickly the cells may develop into an invasive cancer (or how likely it is that the DCIS will come back after surgery). Low-grade DCIS has the lowest risk of developing into an invasive cancer, and high-grade carries the greatest risk | | NR | Diagnosis: Diagnostic tests: mammogram After having a routine mammogram on the NHS Breast Screening Programme, most women will receive a results letter about two weeks later. About 96 per cent of women have a normal result from their first mammogram and will be invited for screening again three years later. About four in every 100 women screened, though, are called back for further tests because of an abnormality seen on the mammogram. Diagnostic tests: ultrasound scans An ultrasound scan uses high-frequency sound waves to produce a picture of the breast.Diagnostic tests: biopsy: A biopsy involves taking a small sample of cells or tissue from the breast and looking at the sample under a microscope. Needle (core) biopsy: A needle or core biopsy involves a doctor using a needle to obtain a sample of tissue to further investigate abnormalities found on screening mammograms and to obtain a definite diagnosis. Fine Needle Aspiration (name mentioned only).Currently, women with DCIS are treated either by wide local excision (WLE) or mastectomy. Wide local excision involves the surgical removal of the affected breast tissue, together with an area (margin) of normal breast tissue around it, to ensure that all affected tissue is removed. WLE is an example of breast-conserving treatment – only the area of DCIS is removed, rather than the whole breast. Mastectomy involves removal of the whole breast and is usually recommended if the DCIS affects a large area of the breast, if it has not been possible to get a clear area of normal tissue around the DCIS by wide local excision, or if there is more than one area of DCIS. Breast reconstruction using an implant. There are two main types of breast reconstruction: reconstruction using a breast implant and reconstruction using a woman’s own tissue. The aim of reconstructive surgery is to replace breast tissue lost during mastectomy or lumpectomy, restoring the breast shape. Breast reconstruction using tissue from your back: an LD flap: This operation involves moving a large muscle (latissimus dorsi) and some overlying fat and skin from the back of the body. The flap and its blood supply are tunnelled under the skin just below the armpit. Breast reconstruction using tissue from your tummy: DIEP flap, SIEA flap, TRAM flap. Or your bottom or thigh: SGAP, IGAP,TMG,TUG.: In this procedure, a breast form is created by taking skin and fat and sometimes muscle from the lower abdomen (tummy). There are three types of breast reconstruction surgery using tissue from the lower abdomen; a DIEP flap, a SIEA flap and a TRAM flap. In a DIEP and SIEA flap only skin and fat is used, but in a TRAM flap muscle is also used. Nipple reconstruction: Mastectomy usually means removal of the whole breast including the nipple and areola (the coloured area of skin around the nipple), but it is possible to have the nipple reconstructed. Nipple reconstruction is usually done a few months after the breast reconstruction to give the new breast time to settle into its final shape and position. Breast prostheses: Women who had a mastectomy without immediate breast reconstruction were given a lightweight foam prosthesis (false breast), which they could put inside their bra. This is sometimes called a cumfie or softie. Radiotherapy for DCIS Radiotherapy treats cancer by using high energy rays to destroy the cancer cells, while doing as little harm as possible to normal cells. Treatment is usually given daily for several weeks as an out-patient. It is often used after surgery for breast cancer, though may occasionally be used before or instead of surgery. Chemotherapy and DCIS: Women who have been diagnosed with DCIS do not need to have chemotherapy. Hormone therapy for DCIS: Hormonal therapies are commonly prescribed in women with invasive breast cancer because they reduce the risk of breast cancer coming back. | NR | Resources: Breast Cancer Care breastcancercare.org.uk Breast Cancer Care is the UK's leading provider of information, practical assistance and emotional support for anyone affected by breast cancer. The link takes you to their information about DCIS. Complementary and alternative medicine for cancer: 20 questions and answers. Compiled by healthtalk.org and Dr. Andrew Vickers of the Memorial Sloan-Kettering Cancer Center, New York. See also our general resources on Cancer, General health and medicine, Mental health and wellbeing and Practical matters. |
| National Health Service | NR | non-invasive breast cancer, cancer cells | Non-invasive breast cancer: About 1 in 5 women diagnosed with breast cancer through screening will have non-invasive cancer. This means there are cancer cells in the breast, but they are only found inside the milk ducts (tubes) and have not spread any further. This is also called ductal carcinoma in situ (DCIS). | NR | NR | Screening does not prevent you from getting breast cancer….Non-invasive breast cancer: About 1 in 5 women diagnosed with breast cancer through screening will have non-invasive cancer. In some women, the cancer cells stay inside the ducts. But in others they will grow into (invade) the surrounding breast in the future | In some women, the cancer cells stay inside the ducts. But in others they will invade the surrounding breast tissue in the future. | About 1 in 5 women diagnosed with breast cancer through screening will have non-invasive cancer. | Breast screening uses an X-ray test called a mammogram to check the breast for signs of cancer. It can spot cancers that are too small to see or feel. You may be asked if you want to take part in a clinical trial: These are medical research studies. Breast cancer treatment: Whether the cancer is invasive or non-invasive, you will be offered treatment and care from a team of breast cancer specialists. The treatment is likely to include surgery (which may mean a mastectomy), radiotherapy, hormone therapy and possibly chemotherapy. These treatments can cause long-term side effects. | NR | Who can I contact if I have a question? If you have questions about screening, please contact your local breast screening unit. If you would like to talk to someone about whether to have breast screening, your GP can help. Together, you can weigh up the possible benefits and risks, to help you decide. For more information about breast screening visit: www.nhs.uk/breast To find details of your local breast screening unit visit: www.nhs.uk/breastscreening It’s your choice whether to have screening or not. If you decide you don’t want any more invitations, you can opt out. Visit: www.gov.uk/phe/screening-opt-out to find out how. For information on how NHS screening programmes use patient information safely and securely visit: www.gov.uk/phe/screening-data |
| Alaska Breast Care and Surgery | NR | Stage 0, pre invasive breast cancer | Ductal Carcinoma in-situ, DCIS, is considered a Stage 0 or pre-invasive breast cancer. What does it mean to be in-situ or pre-invasive? It means that when the pathologist looks at the cells under the microscope, they do not see any evidence that the cells have the ability to invade into the surrounding tissue. The cells originate inside the milk duct and are all still confined within the milk duct. | Get rid of bad cells before they develop ability to spread. | NR | NR | NR | NR | Treatment for DCIS: The surgical treatment for DCIS are similar to invasive breast cancer. Partial mastectomy (sometimes called lumpectomy) is generally encouraged as long as the surgeon feels that breast will tolerate the extent of the resection with an adequate cosmetic outcome and there are no contraindications for breast preservation. Mastectomy is reserved for when there is a large extent of disease or when the patient’s preference is for mastectomy. Reconstruction is always encouraged. We urge all our patients to meet with Plastic Surgery, even those who think they would not want a reconstruction, just to make sure that they understand all the options out there. We also want everyone to be reassured by the skill of our Anchorage based Plastic Surgeons. | NR | NR |
| American Cancer Society | NR | Cancer, non-invasive, pre-invasive breast cancer | Ductal carcinoma in situ (DCIS) means the cells that line the milk ducts of the breast have become cancer, but they have not spread into surrounding breast tissue. DCIS is considered non-invasive or pre-invasive breast cancer. DCIS can’t spread outside the breast, but it still needs to be treated because it can sometimes go on to become invasive breast cancer (which can spread). | DCIS can progress into invasive breast cancer, treatment aims to prvent progression. DCIS can’t spread outside the breast, but it still needs to be treated because it can sometimes go on to become invasive breast cancer (which can spread). | NR | Hormone therapy after surgery can lower the risk of DCIS or invasive cancer developing in either breast...If BCS is done, it is usually followed by radiation therapy. This lowers the chance of the cancer coming back in the same breast (either as more DCIS or as an invasive cancer). | Hormone therapy and radiation therapy after surgey lowers risk of DCIS recurrence as invasive cancer or more DCIS | NR | Breast-conserving surgery (BCS) In breast-conserving surgery (BCS), the surgeon removes the tumor and a small amount of normal breast tissue around it. Lymph node removal is not always needed with BCS, but it may be done if the doctor thinks the area of DCIS might also contain invasive cancer. The chances an area of DCIS contains invasive cancer goes up with tumor size and how fast the cancer is growing. . If lymph nodes are removed, this is usually done as a sentinel lymph node biopsy (SLNB). If BCS is done, it is usually followed by radiation therapy. This lowers the chance of the cancer coming back in the same breast (either as more DCIS or as an invasive cancer). BCS without radiation therapy is not a standard treatment, but it might be an option for certain women who had small areas of low-grade DCIS that were removed with large enough cancer-free surgical margins. Mastectomy: Simple mastectomy (removal of the entire breast) may be needed if the area of DCIS is very large, if the breast has several areas of DCIS, or if BCS cannot remove the DCIS completely (that is, the BCS specimen and re-excision specimens still have cancer cells in or near the surgical margins). Many doctors will do a SLNB along with the mastectomy. This is because if an area of invasive cancer is found in the tissue removed during a mastectomy, the doctor won’t be able to go back and do the SLNB later, and so may have to do a full axillary lymph node dissection (ALND). Hormone therapy after surgery If the DCIS is hormone receptor-positive (ER-positive or PR-positive), adjuvant treatment with tamoxifen (for any woman) or an aromatase inhibitor (for women past menopause) | NR | NR |
| California Department of Health Care Services | Your Health care team: No one health care professional is able to provide all of the types services you may need. Here are some of the experts who could become part of your healthcare team. You will find their descriptions in the Words to Know section in the back of this booklet. Anesthesiologist, Case Manager, Clinical Nurse Spcialist, Lymphedema Therapist, Occupational Therapist, Oncologist, Oncology Nurse, Pathologist, Patient Advocate, Patient Navigator, Physical Therapist Plastic Suregon, Primary Care Provider, Psychologist, Radiation Oncologist, Radiation Therapist, Radiologist, Radiology Technologist, Registered Dietician, Social Worker, Surgeon. | non-invasive cancer | Ductal Carcinoma in Situ (DCIS) ia a non-invasive cancer that is found in the milk duct of the breast and has not spread outside the duct. However, some cases of DCSI will eventually change into invasive breast cancers if left untreated. Since it is not known which ones will change, surgical removal of the cancer followed by radiation therapy is almost always recommended. About 20% of newly diagnosed breast cancers are DCIS. | However, some cases of DCSI will eventually change into invasive breast cancers if left untreated. Since it is not known which ones will change, surgical removal of the cancer followed by radiation therapy is almost always recommended. | NR | The risk of invasive cancer or recurrence of DCIS is very low ....Recurrent Breast Cancer: Breast Cancer that comes back after initla treatment is called recurrent breast cancer. Although this can happen at any time, most recurrences happen within 3 to 5 years. Breast cancer may recur locally, regionally, or, it may recur in a more distant part of the body. Treatment for recurrent breast cancer depends on the place of recurrence and the initial treatment | Risk of invasive cancer or DCIS recurrence is very low. Breast cancer may recur locally, regionally or distally. Treatment for recurrent breast cancer depends on location and initial treatment used. | Most reccurences happen within 3-5 years | Treatment by Stage: Stage 0 (DCIS) - breast conserving surgery followed by radiation - breast conserving surgery (also called lumpectomy or partial masectomy) removes the cancer along with a small rim of normal, healthy tissue OR - total masectomy - removes the breast inclusing the nipple, but not the underlying lymph nodes, OR - breast-conserving surgery without radiation (*for a limited subgroup of women)* Systemic treatment after surgery may include the folowing: - hormone therapy *(for women with hormone receptor-positive breast cancer) -* Hormone therapy is a type of systemic therapy that works by blocking or lowering the amount of hormones in the body. Clinical Trials - are research studies that test new drugs and new medical devices. | Follow Up Care: The purpose of follow up care is to monitor and manage any long term or late effects of treatment and to check for any signs that the cancer may have returned. Women who have finished breast cancer treatment will still see their doctor regularly. For the first three years after treatment, you will ussually see your doctor every 3 to 6 months, then every 6 to 12 months for the next two years, than once a year...Thorough clincal breast exam, including feeling lymph nodes, looking for any visual changes, MRI may be recommedned. Immediately report: - a new lump in breast or on chest wall - a new lump in the armpit or neck - a chnage in the shape of the breast - a skin rash, swelling, or chnage in the colour of the skin over the breast or chest - spontaneous nipple discharge | Question Prompt Lists: (ex. Questions to ask your Doctor… - What type of breast reconstruction do you recommend? Why? - When do you recommned that I begin breast reconstruction? - What results are realistic for me? - Will there be scars? Where? How large? For More Information: This booklet is a starting point for learning about your treatment options. It may not include all treatments or tell you all you need to know about side effects and possible problems. F or more information you can contact the American Cancer Society or the National Cancer Institute. American Cancer Society (ACS): Call 1-800-277-2345 or go online at www.cancer.org. National Cancer Insitute: Call 1-800-422-6237 or go online at www.cancer.gov. |
| Living Beyond Breast Cancer | Your Medical Team…or “Who Are All These People?” Your healthcare providers are a key part of your support team. Many hospitals take a team-based approach to care, meaning your doctors work together to follow your case and meet regularly to discuss your treatment. A radiologist reads pictures of organs and tissues and looks for disease. The radiologist reviews images from mammograms, sonograms, ultrasounds, MRIs, CAT scans, bone scans and PET scans. A pathologist diagnoses disease by looking at tissues under a microscope and figuring out the specific type of breast cancer. A surgical oncologist removes cancer during surgery. Surgical oncologists are general surgeons who receive special training in the diagnosis and surgical treatment of cancer. A medical oncologist diagnoses and treats cancer with medicines such as chemotherapy, hormonal therapy and targeted therapy. A nurse navigator is a nurse who guides you and your caregivers through the healthcare system so you can get the care you need to make informed decisions about treatment. A radiation oncologist treats cancer with radiation therapy. A genetic counselor is trained to talk to you about your family history of cancer. A reproductive endocrinologist is an expert on fertility, a woman’s ability to have children. A plastic or reconstructive surgeon reshapes or rebuilds the breast after surgery. A primary care physician provides you with your regular medical care and may manage your care with your other doctors. A medical oncology nurse is a registered nurse with special training in the medical and emotional needs of people with cancer. A surgical oncology nurse is a registered nurse with special training in the surgical treatment and emotional needs of people with cancer. A nurse practitioner is an advanced practice registered nurse with special training in the treatment and emotional needs of people with cancer. A social worker or counselor talks with you and your family about your emotional needs and helps you find support services. A physician assistant is a licensed health provider who can diagnose and treat cancer with a doctor’s oversight. A patient navigator is a nurse, social worker or person who helps you and your loved ones quickly get access to the tools you need to get treatment and keep your quality of life. A physical therapist performs and teaches exercises to help you maintain strength before starting treatment and to improve recovery | cancer cells, Stage 0 breast cancer | When cancer cells stay inside and fill the walls of the ducts, this is called in situ breast cancer or ductal carcinoma in situ (DCIS). Stage 0 breast cancer is noninvasive, or DCIS. It is confined to the ducts of the breast | Remove tumour and protect body from future invasive cancer. Two types of therapy. Local therapy: Control DCIS in breast. Systemic therapy: Get rid of cancer cells that may have spread. | Breast cancer treatment affects many other areas of your life, both physically and emotionally. Whatever your normal daily life, you may need or want to take a break, cut back or change your responsibilities. The costs of treatment may impact your financial outlook. Most of us are used to caring for others, whether that means earning money to support our families, managing a busy household or giving emotional support. Whatever way you look at it, breast cancer puts many of us in the unfamiliar position of asking others for help. Where Can You Find Support?: You may wish to look for support outside of family and friends. Your treatment center is a great place to start. Let your doctors and nurses know how you’re feeling. Ask to speak with an oncology social worker or counselor. Many hospitals have libraries just for people with cancer. | People with DCIS are at increased risk of developing invasive cancers | People with DCIS are at increased risk of developing invasive cancers | NR | To find out, your doctor may do a sentinel lymph node biopsy (surgery to check for cancer in several lymph nodes under your arm) and blood tests, a chest x-ray, CAT scan, MRI, bone scan or PET scan. Your doctor will get answers to other questions by looking at tests you already had, including the mammogram, ultrasound or biopsy. Other tests may be done on pieces of tissue taken from your breast during the biopsy. Taken together, the results of these tests will create a “profile” of the cancer that will help you and your treatment team decide on next steps. In some cases, you may have a single treatment option, or you may have one option among several that makes most sense for you. Surgery: Almost everyone diagnosed with breast cancer will have some kind of surgery. The goal of surgery is to remove the cancer from your breast. There are two types of breast surgery, breast conservation and mastectomy. In breast conservation, the surgeon performs a lumpectomy (also called a partial mastectomy or segmental excision), which is followed by radiation treatment to the remaining breast tissue.If you cannot have breast conservation because of the size or extent of the tumor and you wish to do so, you may be able to get chemotherapy, hormonal therapy, or targeted therapy before surgery to shrink the tumor, called neoadjuvant therapy. Another option is to use a prosthesis, an artificial breast form that fits into your bra, instead of having reconstructive surgery. Radiation Therapy: Radiation treatment is another local therapy. The goal of radiation therapy is to kill any cancer cells left in areas of your body at high risk for return of cancer. It helps protect you from the breast cancer coming back in the same place (local recurrence). Genomic Tests: Your doctor may also recommend the test if you have DCIS. This is because a new method of testing using the Oncotype DX technology may help your healthcare team predict whether DCIS has a low, intermediate, or high risk of recurrence, called a DCIS Score. Chemotherapy:In some cases, the risk the cancer will come back is not clearly high or low, and you may have a choice about whether to have chemotherapy. If so, explore with your doctor the benefits and risks of chemotherapy. Targeted Therapy: Targeted therapies are medicines that fight cancer by finding and killing only cancer cells. Hormonal Therapy: Some cancers rely on the hormones estrogen and progesterone to grow and survive. (Tamoxifen, Aromatase Inhbitiors, Ovarian Suppression or Ablation, Oophorectomy). Complementary and Integrative Medicine (CIM), once called complementary and alternative medicine (CAM), has become a popular way to manage treatment side effects. Complementary medicine brings together many types of treatments, including acupuncture, Qigong, the creative arts, and yoga. | NR | List of Resources: ex. People of Color  Asian and Pacific Islander American Health Forum: (415) 954-9988, apiahf.org  Dia de la Mujer Latina: (281) 489-1111, diadelamujerlatina.org  Intercultural Cancer Council and Caucus: (713) 798-4614, iccnetwork.org  Native American Cancer Research Corporation: (303) 838-9359, natamcancer.org  Sisters Network: (866) 781-1808, sistersnetworkinc.org Learning More About Your Pathology Report Breastcancer.org: Your Pathology Report, breastcancer.org/symptoms/diagnosis/getting_path_report.jsp  Oncolink.org: search for “breast cancer pathology report” Susan G. Komen: What Is a Pathology Report?, (877) 465-6636, ww5.komen.org/BreastCancer/WhatisaPathologyReport.html Specific Cancer Diagnoses or Situations  HER2-positive: her2support.org  Inflammatory Breast Cancer: eraseibc.org, ibcsupport.org, ibcresearch.org  Premenopausal: livestrong.org/we-can-help/fertility-services, youngsurvival.org  Hereditary: facingourrisk.org Triple-Negative Breast Cancer: tnbcfoundation.org, Question Prompt List: 10 Questions to Ask Your Doctor About Treatment Why are you recommending this treatment for me? What are the pros and cons of this treatment? What are the possible short-term side effects of these treatments? What are the possible long-term side effects? Can I take part in any research trials? |
| National Health Service | NR | non-invasive breast cancer | Non-invasive breast cancer (carcinoma in situ) – found in the ducts of the breast (ductal carcinoma in situ, DCIS) and hasn't developed the ability to spread outside the breast. It's usually found during a mammogram and rarely shows as a breast lump. | NR | Breast cancer can affect your daily life in different ways, depending on what stage it's at and the treatment you're receiving: How women cope with their diagnosis and treatment varies from person to person, but there are several forms of support if you need it. Not all of them work for everybody, but one or more of them should help. | NR | NR | NR | General Treatment: Diagnosis: Mammogram and breast ultrasound. If you have symptoms and have been referred to a specialist breast unit by your GP, you'll probably be invited to have a mammogram, which is an X-ray of your breasts. You may also need an ultrasound scan. Biopsy: A biopsy is where a sample of tissue cells is taken from your breast and tested to see if it's cancerous. Needle aspiration may be used to test a sample of your breast cells for cancer or drain a small fluid-filled lump (benign cyst). Your doctor may suggest that you have a guided needle biopsy, usually guided by ultrasound or X-ray, but sometimes MRI, to obtain a more precise and reliable diagnosis of cancer. Vacuum-assisted biopsy, also known as mammotome biopsy, is another type of biopsy. Scans and X-rays. Computerised tomography (CT) scans or chest X-ray and liver ultrasound scans may be needed to check whether the cancer has spread. An MRI scan of the breast may be needed to clarify the results or assess the extent of the condition within the breast. Treatment: Surgery: There are 2 main types of breast cancer surgery: breast-conserving surgery – the cancerous lump (tumour) is removed mastectomy – surgery to remove the whole breast In many cases, a mastectomy can be followed by reconstructive surgery to try to recreate a bulge to replace the breast that was removed. Studies have shown that breast-conserving surgery followed by radiotherapy is as successful as total mastectomy at treating early-stage breast cancer.Breast-conserving surgery: Breast-conserving surgery ranges from a lumpectomy or wide local excision, where just the tumour and a little surrounding breast tissue is removed, to a partial mastectomy or quadrantectomy, where up to a quarter of the breast is removed. Chemotherapy: Chemotherapy involves using anti-cancer (cytotoxic) medication to kill the cancer cells. It's usually used after surgery to destroy any cancer cells that haven't been removed. This is called adjuvant chemotherapy. After having breast-conserving surgery, you'll usually be offered radiotherapy to destroy any remaining cancer cells. Mastectomy: A mastectomy is the removal of all the breast tissue, including the nipple. Reconstruction: Breast reconstruction is surgery to make a new breast shape that looks like your other breast as much as possible. Lymph node surgery: To find out if the cancer has spread, a procedure called a sentinel lymph node biopsy may be carried out. The sentinel lymph nodes are the first lymph nodes that the cancer cells reach if they spread. They're part of the lymph nodes under the arm (axillary lymph nodes). The sentinel lymph nodes are examined in the laboratory to see if there are any cancer cells present. This provides a good indicator of whether the cancer has spread. If there are cancer cells in the sentinel nodes, you may need further surgery to remove more lymph nodes from under the arm. Radiotherapy: Radiotherapy uses controlled doses of radiation to kill cancer cells. It's usually given after surgery and chemotherapy to kill any remaining cancer cells. If you need radiotherapy, your treatment will begin about a month after your surgery or chemotherapy to give your body a chance to recover. You'll probably have radiotherapy sessions 3 to 5 days a week, for 3 to 6 weeks. Each session will only last a few minutes. The type of radiotherapy you have will depend on your cancer and the type of surgery you have. Some women may not need to have radiotherapy at all. Chemotherapy: Chemotherapy involves using anti-cancer (cytotoxic) medication to kill the cancer cells. Hormone treatment: Some breast cancers are stimulated to grow by the hormones oestrogen or progesterone, which are found naturally in your body. | Follow-up: After your treatment has finished, you'll be invited for regular check-ups, usually every three months for the first year. If you've had early breast cancer, your healthcare team will agree a care plan with you after your treatment has finished. This plan contains the details of your follow-up. You'll receive a copy of the plan, which will also be sent to your GP. During the check-up, your doctor will examine you and may carry out blood tests or X-rays to see how your cancer is responding to treatment. You should also be offered a mammogram every year for the first five years after your treatment. | Want to know more? Breast Cancer Care: lymphoedema Breast Cancer Care: your operation and recovery (PDF, 148kb) Cancer Research UK: breast cancer follow-up Macmillan Cancer Support: follow-up after breast cancer treatment |
| Worcester Breast Surgery | NR | early form of breast cancer, pre invasive cancer, intra-ductal cancer or non-invasive cancer | DCIS is a VERY early form of breast cancer, where the cancer cells have developed within the milk ducts but remain there (so called ‘in situ’) as the cells don't have the ability to spread outside the ducts into the surrounding breast tissue or to other parts of the body. So it is usually described as a pre-invasive, intraductal or noninvasive cancer. Both men and women can develop DCIS, however it is very rare in men. As a result of being confined to the breast ducts, a diagnosis of DCIS has a very good outlook. | NR | NR | Women with DCIS have an excellent prognosis. By treating DCIS in a specialist centre you ensure your health is in the best possible hands. Following treatment for DCIS most women are offered annual screening mammograms for 5 years or to age 50 to monitor both the treated breast and the opposite healthy breast. By definition, there is no risk of distant recurrence since the cancer is noninvasive. | Women with DCIS have an excellent prognosis.There is no risk of distant recurrences since the cancer is noninvasive. | NR | How is DCIS diagnosed? If the radiologist (X-ray doctor) who read your mammogram suspects you have DCIS, he or she will arrange for you to have a mammographically guided (stereotactic) biopsy. The biopsy can often be done the same day, you will be given an idea of the most likely diagnoses and the biopsy report will follow within a few days.What is the treatment for DCIS?: Local excision with radiation therapy. Most patients have great success rates having a wide local excision (lumpectomy or breast conserving treatment ) and subsequent X-ray treatment to the breast. Rachel has great experience treating women diagnosed with DCIS through the breast screening programme (> 40 cases/year) with a variety of “oncoplastic” operations aimed to minimise any change in breast shape or size whilst giving first class cancer treatment. • Mastectomy. Some women have more extensive DCIS where a mastectomy may be the more appropriate surgical treatment instead of a lumpectomy. This would generally be accompanied with a lymph node biopsy from the armpit at the same time and is unlikely to require any additional radiotherapy afterwards. Mastectomy can often be accompanied by immediate breast reconstruction should this be required. Rachel can offer a range of immediate or delayed breast reconstruction techniques and works closely with several plastic surgeons if additional input is required. • Chemotherapy. Chemotherapy is not needed for DCIS, since the disease is noninvasive. • Hormonal Therapy. Hormonal therapy is only occasionally recommended as part of a clinical trial if the DCIS expresses the oestrogen receptor on the surface of the cells. | NR | NR |
| The Newcastle upon Tyne Hospitals | NR | NR | NR | NR | If you have any worries or concerns in between your appointments please do not hesitate to contact your GP or Nurse Specialist who will advise you | NR | NR | NR | Diagnosis: DCIS is most commonly detected with a mammogram when there are no physical signs in the breast. When your surgery and any radiotherapy are finished you will have an appointment with the Radiotherapy Team after around two months. Only a guide for treatment completion of DCIS, does not go into detail regarding treatment options, only mentions a few briefly. | QI. What about hospital appointments? A1. When your surgery and any radiotherapy are finished you will have an appointment with the Radiotherapy Team after around two months. You will have a further appointment with the surgical team at about six months. This allows the medical teams involved in your care to check how you are and make sure you have recovered from treatment. Q2. How often will I have a mammogram? A2 You will be invited for a mammogram every year for a total of five years unless you are under 42 years old in which case you will have annual mammograms until you reach the age of routine breast screening (usually from age 47). Q3. What symptoms should I report? A3. You should remain breast aware and remember the five point code:- · Changes in breast shape and size · Any puckering or dimpling of the skin · Discomfort or pain that is different from normal · Any new lumps, thickening or bumpy areas in the breast or armpit · Nipple changes or nipple discharge (not milky). | Contact Details Nurse Specialists: Telephone: 0191 2820207 or 0191 2820208 Office hours: 8.30am-4.30pm (Monday to Friday) Email: BreastCareNurses@nuth.nhs.uk Alternative contacts: The Patient Advice and Liaison Service (PALS) can offer on-the-spot advice and information about the NHS. You can contact them on freephone 0800 032 02 02 or email northoftynepals@nhct.nhs.uk Useful websites Further information is available on the hospital website: www.newcastle-hospitals.org.uk |
| Princess Margaret Hospital – University Health Network | NR | early form of breast cancer | Ductal carcinoma in situ is an early form of breast cancer. DCIS is a type of tumour that only grows in the milk ducts of the breast. It does not spread out into the rest of the body. | NR | NR | If you had a lumpectomy, you will need radiation treatment after surgery. It will help lower the chance that the DCIS will come back. It will also help lower the chance of you getting breast cancer that could spread (invasive cancer) in the future. | Radiation treatment lowers the chance of DCIS reccurance, and spread (invasive cancer) in the future. | NR | DCIS is often found during a mammogram (x-ray exam of the breast). On a mammogram DCIS tends to look like micro calcifications (small calcium deposits). It can be hard to spot. If your doctor thinks you have DCIS, they will ask you to have a biopsy. This is when the doctor takes small samples of tissue from the breast for testing. .Surgery: Everyone who has DCIS will need surgery. There are two types of surgery: 1. Lumpectomy (removing a part of the breast) 2. Mastectomy (removing the whole breast). Your doctor will discuss with you which option is right for you. If not all the DCIS has been removed, you may need a second surgery to remove the remaining DCIS. Radiation treatment: This treatment uses high-energy radiation to kill cancer cells. It works by damaging and later killing the cells in the treatment area. If you had a lumpectomy, you will need radiation treatment after surgery. It will help lower the chance that the DCIS will come back. It will also help lower the chance of you getting breast cancer that could spread (invasive cancer) in the future. If you had a mastectomy, you will most likely not need radiation treatment after surgery. You may need radiation treatment if the tumour has “close margins” or if the area of DCIS is very large. 2. Endocrine therapy: Based on current information, the use of hormoneblocking (also known as endocrine or hormone) therapy is not a standard treatment for DCIS. In some cases of DCIS, the use of endocrine therapy may be suggested. | Will I have follow up appointments? Yes, you will need to book a follow up with a doctor once a year. During the appointment, you will get: • a mammogram or other type of breast imaging • a breast exam. Your surgeon will tell you how to book appointments, and which doctor(s) you will meet for the follow-ups. | NR |
| American Cancer Society | NR | abnormal cells | DCIS refers to abnormal cells that replace the normal epithelial cells of breast ducts, but are still within the tissue layer of origin; under a microscope, these cells appear similar to those of invasive breast cancers. | If untreated, DCIS can become invasive and is considered cancer precursor. Treatment of DCIS prevents progression to invasive cancer. | NR | Long-term followup studies of patients with carcinoma in situ also find that even without treatment, not all patients develop invasive cancer…studies also show that some women treated for DCIS might not have developed an invasive breast cancer in the absence of treatment. | Long-term studies of patients with carcinoma in situ find that even without treatment, not all patients develop invasive cancer. Studies also show some women treated for DCIS might not have developed invasive breast cancer in the absence of treatment. | NR | Although DCIS can present as a palpable mass, it is most often detected by a mammogram, where it commonly is identified by the appearance of microcalcifications (tiny bits of calcium that appear as clustered white dots). Overdiagnosis and overtreatment of DCIS (terms that are usedto describe diagnosis and treatment of diseases that would have gone undetected in the absence of screening) are of concern because the diagnosis, treatment, and follow-up can affect longterm health and quality of life. Treatment for DCIS: Treatment for DCIS usually involves either breast-conserving surgery (BCS) with radiation therapy or mastectomy. BCS removes a part of the affected breast, including the area where DCIS is found, along with a margin of healthy tissue. If the removed tissue is later found to also contain invasive cancer, staging of the axillary (underarm) lymph nodes is needed. This is most often done using a minimally invasive staging procedure called a sentinel lymph node biopsy. Radiation therapy is recommended for most women who have BCS because randomized trials show strong and consistent evidence that radiation therapy after BCS approximately halves the rate of recurrence in the affected breast. Some women with unilateral DCIS choose to have bilateral mastectomy to prevent cancer in the unaffected breast. For women with ER+ DCIS, hormonal therapy with tamoxifen is associated with a significantly decreased risk of invasive cancer and DCIS in either breast. For example, the Oncotype DCIS Score, which measures the expression of a group of cancer genes in the tumor tissue, has been developed and validated as a predictor of recurrence in selected patients treated with BCS without radiation. | NR | Reference List: Ex. 1. Partridge AH, Elmore JG, Saslow D, McCaskill-Stevens W, Schnitt SJ. Challenges in ductal carcinoma in situ risk communication and decision-making: report from an American Cancer Society and National Cancer Institute workshop. CA Cancer J Clin. 2012;62: 203-210. 2. Elmore JG, Fenton JJ. Ductal carcinoma in situ (DCIS): raising signposts on an ill-marked treatment path. J Natl Cancer Inst. 2012;104: 569-571. |
| Macmillan Cancer Support | Multidisciplinary team (MDT): A team of specialists will meet to discuss and decide on the best treatment for you. After the MDT meeting, your doctor will talk to you about your treatment choices. This multidisciplinary team (MDT) will include: • a specialist breast surgeon • a breast reconstruction surgeon • a specialist breast care nurse who gives information and support • clinical oncologists – doctors who specialise in treating cancer using radiotherapy and drug treatments (such as hormonal therapy) • a radiologist who reads mammograms and analyses scans • a pathologist who examines cells under the microscope and advises on the type and extent of the cancer. The MDT may also include other healthcare professionals, such as a research nurse, a physiotherapist, counsellor, psychologist or a social worker. | earliest form of breast cancer, non-invasive, Stage 0 | DCIS is the earliest possible form of breast cancer. DCIS is non-invasive. This means that breast cancer cells are in the milk ducts, but they haven’t spread into (invaded) surrounding breast tissue. DCIS is described as stage 0. This is the earliest stage and means there is no invasive breast cancer. DCIS can be any size but will always be stage 0. In DCIS, the grade of the cells is important. It shows how likely DCIS is to come back in the breast, or to develop into an invasive cancer. There are three grades: Low-grade DCIS – The cells look similar to normal breast cells and usually grow slowly. The cancer cells are less likely to spread into surrounding tissue. Moderate- or intermediate-grade DCIS – The cells look more abnormal and grow slightly faster than low-grade DCIS. High-grade DCIS – The cells look quite different from normal breast cells and grow more quickly. | Without treatment DCIS may become invasive cancer and spread. Treatment reduces risk of recurrence and progression to invasive cancer. If DCIS isn’t treated, it may over time spread into (invade) the breast tissue surrounding the ducts to become an invasive breast cancer | NR | Having DCIS means you have a slightly higher risk of getting cancer elsewhere in the same breast or in your other breast....If DCIS comes back: After treatment, the risk of DCIS coming back or of an invasive cancer developing is low. If any new problems develop, they will usually be picked up very early. If DCIS comes back or an invasive cancer develops in the same breast, you’ll usually be advised to have a mastectomy. If you haven’t had radiotherapy, it might be possible to remove the area with surgery and then have radiotherapy. Treatment for DCIS that comes back or for early invasive breast cancer that develops after treatment for DCIS is usually very successful. | Having DCIS means you have a slightly higher risk of getting cancer elsewhere in the same breast or in the other breast. After treatment, the risk of DCIS coming back or of invasive cancer developing is low. | NR | How DCIS is diagnosed: Mammogram A mammogram is a low-dose x-ray of the breast, which can detect changes in the breast tissue. Breast ultrasound: An ultrasound scan uses sound waves to build up a picture of the breast tissue. It can show whether an abnormal area is solid. Breast biopsy: A biopsy is needed to diagnose DCIS. The doctor injects a local anaesthetic and then takes a small piece of tissue or cells (biopsy) from any abnormal areas.(made of cells) or is a fluid-filled cyst. Core needle biopsy with mammogram: A mammogram may be used to guide the biopsy. This is known as a stereotactic core needle biopsy. Ultrasound-guided needle biopsy: Sometimes an ultrasound scan is used to show where the abnormal area is. Vacuum-assisted biopsy (VAB): A mammogram or ultrasound helps the doctor to guide this biopsy needle to the correct area of the breast. Clip insertion: When a core biopsy is performed, a tiny metal clip is often placed in the area. Excision biopsy: The surgeon makes a cut in the skin of the breast and takes a biopsy of the breast tissue. Wire localisation:  Sometimes an x-ray or ultrasound is used to guide a fine wire into the breast. Examining the tissue under a microscope (pathology). Surgery: Surgery is the main treatment for DCIS. Wide local excision (WLE):  The surgeon removes the DCIS and some of the normal-looking tissue around it (a clear margin). Clear margins: After surgery, the pathologist examines the tissue in the edges of the tissue (margin) around the DCIS. Mastectomy (removing the breast): Breast surgeons will usually try to conserve a woman’s breast whenever possible. But sometimes they may recommend a mastectomy. This may be when the DCIS: • covers a wide area • is in more than one part of the breast and the affected areas are not small. Sentinel lymph node biopsy (SLNB): During surgery, your surgeon may remove a few lymph nodes from the armpit to see if they contain cancer cells. Breast reconstruction: If you are having a mastectomy, your surgeon will usually ask if you want a new breast shape made at the same time (immediate breast reconstruction). Breast prosthesis: If you have a mastectomy and don’t have immediate breast reconstruction, your nurse will give you a soft, lightweight prosthesis (false breast) to wear inside your bra. Radiotherapy: uses high-energy rays to destroy cancer cells, while doing as little harm as possible to normal cells. Hormonal therapies: reduce the level of oestrogen in the body, or prevent it from attaching to the cancer cells (Tamoxifen, Aromatase Inhibitors). Research – clinical trial: Cancer research trials are carried out to try to find new and better treatments for cancer. | Follow-up: After treatment finishes, you’ll have regular check-ups. If you are below screening age, you will have yearly mammograms until you enter an NHS Breast Screening Programme. If you are already having screening, you will have a mammogram every year for 5–10 years and then go back to three-yearly screening. If you are over the screening age, you can choose to have regular screening. You won’t receive an invitation letter, but you can contact your local screening clinic to arrange it. After your surgery or radiotherapy, your follow-up appointments may be every few months at first, but eventually are likely to be once a year. Appointments are a good opportunity for you to talk to your doctor or nurse about any concerns you have. But if you notice any new symptoms between appointments, you can contact your doctor or nurse for advice. After treatment: You’ll probably be keen to get back to the things you did before being diagnosed. But you may still be coping with some side effects of treatment and with some difficult emotions. It’s important to talk about any concerns or questions you have with your cancer specialist and breast care nurse. After treatment, you may want to know what to expect, whether there’s anything you should avoid, how to make the most of your health and where to get support. Effects on your sex life: DCIS, its treatments and side effects may affect your feelings about yourself as a woman and your sex life. Contraception: Your doctor will advise you not to use contraception that contains hormones as these can slightly increase the risk of breast cancer. Hormone replacement therapy (HRT): Doctors don’t recommend hormone replacement therapy (HRT) because it contains oestrogen, which could encourage breast cancer cells to grow. Lymphoedema: Lymphoedema is a swelling of the arm that sometimes happens after surgery or radiotherapy to the lymph nodes in the armpit. Making healthy choices: After treatment, some women choose to make some positive lifestyle changes. Keep to a healthy weight: There’s some evidence that keeping to a healthy weight after the menopause may help reduce the risk of breast cancer coming back. Stick to sensible drinking: Stick to sensible drinking guidelines, which recommend that women drink less than two units a day or 14 units a week. Stop smoking: If you’re a smoker, giving up smoking is the healthiest decision you can make. Get physically active: Being physically active helps to keep your weight healthy and can reduce stress and tiredness. Reduce stress in your life: Being diagnosed with DCIS can be a stressful time in your life...Share your experience: When treatment finishes, you might find it helps to talk about it with other people and share your thoughts, feelings and advice. Work You may need to take time off work during your treatment and for a while afterwards..Financial help and benefits: If you are struggling to cope with the financial effects of cancer, help is available. | Contact Info: For cancer support every step of the way, call Macmillan on 0808 808 00 00 (Mon–Fri, 9am–8pm) or visit macmillan.org.uk...Your notes and questions section…Support groups: Whether you are someone living with cancer or a carer, we can help you find support in your local area, so you can speak face to face with people who understand. Find out about support groups in your area by calling us or by visiting macmillan.org.uk/ selfhelpandsupport. Online community: Share your experiences, ask questions, or just read through people’s posts at macmillan.org.uk/ community. Help with money worries: Financial advice: Our financial guidance team can give you advice on mortgages, pensions, insurance, borrowing and savings. Help accessing benefits:Our benefits advisers can offer advice and information on benefits, tax credits, grants and loans. Macmillan Grants: Macmillan offers one-off payments to people with cancer. Other useful organisations: Ex. Breakthrough Breast Cancer Weston House, 246 High Holborn, London WC1V 7EX Tel 08080 100 200 Email supporterservices@breakthrough.org.uk www.breakthrough.org.uk Committed to fighting breast cancer through research and awareness. Breakthrough Breast Cancer Scotland 38 Thistle Street, Edinburgh EH2 1EN Tel 08080 100 200 Email scotlandinfo@ breakthrough.org.uk |
| Westmead Breast Cancer Institute | NR | early form of breast cancer, pre-cancer | Ductal carcinoma in situ (DCIS) occurs when some of the cells that line a group of milk ducts within your breast have started to become cancer cells. As these cells are contained within the ducts and have not spread into the surrounding breast tissue (in situ), there is very little chance that the cells can spread into the lymph nodes or anywhere else in the body. This can be considered a very early form of breast cancer. It is sometimes also referred to as ‘pre-cancer’. | The cells in DCIS are cancer cells. Without treatment, DCIS may become invasive and spread. | Who can I speak to? Many women find treatment for DCIS difficult to comprehend (and sometimes overwhelming) as they are facing treatments similar to women with invasive breast cancer, yet they have an early stage breast change, often without symptoms. An experienced team is available to help you. Please talk to your doctor, nurse or radiation therapist about anything that is worrying you | Progression: f left untreated, DCIS may develop into invasive breast cancer (cancer that moves beyond the milk ducts). Invasive breast cancer can spread to other parts of your body. | If left untreated, DCIS may develop into invasive breast cancer. Invasive breast cancer can spread to other parts of your body. | NR | DCIS is usually diagnosed on a mammogram where it may be seen as specks of calcium (microcalcification) How is DCIS treated? 1. Wide local excision (breast conservation surgery) and radiotherapy (radiation therapy): Under general anaesthetic, the surgeon removes the area of DCIS and a small area of healthy tissue around it (the ‘surgical margin’). After surgery, radiotherapy (radiation therapy) is given to the breast. This is X-ray treatment that kills cancer cells and significantly reduces the risk of DCIS or invasive breast cancer developing in the remaining breast tissue in the future. Wide local excision without radiotherapy:Wide excision alone may be suitable for some women with small areas of ‘low grade’ DCIS. In most cases surgery is combined with a course of radiotherapy as it has been proven that radiotherapy significantly reduces the chances of DCIS or invasive cancer affecting the breast in the future. 2. Total mastectomy: Under general anaesthetic, the surgeon removes the whole breast and usually the nipple. This is sometimes the only treatment option for large areas of DCIS or cases where small clusters of DCIS are scattered through the breast. 3. Total mastectomy with breast reconstruction Almost all women undergoing mastectomy for DCIS have the option of having breast reconstruction. There are a number of ways that the breast can be reconstructed following mastectomy. Options include reconstruction with a breast implant or a reconstruction with a flap (using your natural tissue from other areas of your body). If the DCIS is away from your nipple you may be able to have a mastectomy that preserves your nipple. Nipple sparing mastectomy: In some mastectomy cases, the nipple can be preserved as long as all of the breast tissue and ducts behind it are removed. 4. Hormonal therapies Hormonal therapies such as tamoxifen and aromatase inhibitors are tablets that are often used in the treatment of invasive breast cancer. Their role in the treatment of DCIS is not completely resolved. Studies show that tamoxifen may reduce the risk of future breast problems after treatment for DCIS. 5. Surgery to remove lymph nodes Most women with invasive breast cancer have surgery to remove some of the lymph glands from their axilla (armpit). We know that if invasive cancer spreads, it is likely to spread to the lymph glands first, so these are removed for testing. When mastectomy is recommended for DCIS, a sentinel node biopsy is frequently recommended | NR | NR |
| Breast Cancer Action | NR | Abnormal cells, non-invasive breast cancer, pre-cancer | DCIS is a condition of abnormal, but not cancerous, cells found in the lining of the milk ducts, and has not spread into nearby tissues. DCIS is the most common type of non-invasive breast ‘cancer’. Non-invasive means that the abnormal cells do not move from where they are found and therefore, DCIS is often referred to as a ‘precancer.’ DCIS represents an intermediate step between normal breast tissue and invasive breast cancer. There are three grades of DCIS: Grade I (low), Grade II (moderate/intermediate), and Grade III (high). The lower the grade, the more closely the DCIS cells resemble normal breast cells and the slower they evolve. | Treatment reduces the risk of recurrence and/or invasive breast cancer. | NR | Although DCIS can be a precursor for development of a subsequent invasive breast carcinoma, this is not the case for most women.ii In fact, if untreated, it is estimated that only about 20-30% of DCIS will go on to become invasive breast cancer. | Although DCIS can be a precursor for development of a subsequent invasive breast carcinoma, this is not the case for most women. | If untreated, it is estimated that only about 20-30% of DCIS will go on to become invasive breast cancer. | A coreneedle biopsy, which uses a hollow-needle that draws out tissue samples, collects cells that are examined under a microscope. Treatment Options: When a woman is diagnosed with DCIS, there are several treatment options. Lumpectomy alone: Lumpectomy is a surgical procedure that removes the breast lump or suspicious tissue and some surrounding tissue. • Lumpectomy plus Radiation: Lumpectomy paired with radiation is used to minimize the chances of having a recurrence of DCIS, by destroying any abnormal cells that have not been removed during surgery. Lumpectomy, plus Radiation and Tamoxifen. Mastectomy Mastectomy is a surgical procedure in which the entire breast, including the nipple, is removed but not the lymph nodes under the arm or the muscle tissue from beneath the breast. Mastectomy plus Tamoxifen. NOTE: Watchful Waiting/Active Surveillance There are some respected breast cancer specialists who believe it’s time to seriously look into what’s known as management of DCIS by active surveillance. | NR | Contact Info: 657 Mission Street, Suite 302 San Francisco, California 94105 Tel: 415.243.9301 Toll free: 1.877.2STOPBC Fax: 415.243.3996 Web: www.bcaction.org www.thinkbeforeyoupink.org Email: info@bcaction.org |
| Breast Cancer Now | Your breast care team: It is recommended that investigations and treatment for breast cancer are carried out at a hospital with a breast unit and a specialist breast care team. This team would normally include: • a breast surgeon and their supporting team • a breast care nurse (or key worker/nurse consultant) • a radiologist (a specialist in the use of x-rays and other imaging methods for diagnosis and treatment) • an oncologist (cancer specialist) • a pathologist (a specialist in the diagnosis of disease by the study of tissues and cells) and laboratory support staff. The team – known as a multidisciplinary team (MDT) – will have experience diagnosing and treating breast cancer | early form of breast cancer, non-invasive breast cancer, pre-invasive or intra-ductal carcinoma | DCIS (ductal carcinoma in situ) – an early form of breast cancer (non-invasive breast cancer) that is confined to the milk ducts and has not spread to neighbouring tissues. The most common type of noninvasive breast cancer is ductal carcinoma in situ (DCIS), also known as pre-invasive or intra-ductal carcinoma. Grading for DCIS is different, and is defined as low, medium or high grade. | DCIS treatment is advised as non-invasive breast cancer can develop ability to spread becoming invasive. | NR | Detecting breast cancer recurrence: Sometimes, a woman’s breast cancer can come back (recur). Recurrences happen when breast cancer cells survive initial treatment and grow into new tumours. There are three types of recurrence: • local recurrence – when the breast cancer comes back in the same place • regional recurrence – when breast cancer comes back in nearby tissues, eg the skin, chest muscles, breast or collar bone area • distant recurrence – when the breast cancer comes back in other areas of the body (metastatic breast cancer). Non-invasive breast cancers sometimes later develop the ability to spread to surrounding tissues, becoming invasive | Recurrence of breast cancer can occur. There are three types of recurrence: • local recurrence – when the breast cancer returns in the same place • regional recurrence – when breast cancer returns in nearby tissues • distant recurrence – when the breast cancer returns in other areas of the body. Non-invasive breast cancers sometimes later develop ability to to spread to surrounding tissues. | NR | Triple assessment: The use of these three tests means an accurate diagnosis can be made promptly. Physical examination: This is an examination of your breasts, your armpits (axillae), the area around your collarbone, and your neck, carried out by a hospital doctor or specialist nurse. Breast imaging: This involves imaging the inside of your breasts using either a mammogram (an x-ray that uses very low doses of radiation) or an ultrasound examination (which uses sound waves). Biopsy: One of two techniques can be used to take a sample of cells (a biopsy) from the lump or area of abnormality in your breast and/or armpit: a core biopsy or FNA.... The treatment offered to you will depend on factors such as the extent of the DCIS, the grade (low, intermediate or high) and where it is within the breast. DCIS is usually treated by surgery, and some women may receive additional treatments, such as radiotherapy. The effectiveness of hormone therapy for DCIS is being assessed in clinical trials. Mastectomy may also be offered for other reasons, including the removal of large areas of early forms of cancer, such as ductal carcinoma in situ (DCIS). Reconstructive Surgery: Women who have a mastectomy may also have breast reconstruction, either at the same time (immediate reconstruction) or at some time in the future (delayed reconstruction). | Follow up: The purpose of follow up is to deal with the side effects of treatments, provide psychological support and to detect and treat any local recurrence of breast cancer…. The length of follow up varies in different centres; it is most often five years. At the end of this time it is important that you continue to attend your mammogram appointments and that you know how to get back in touch with your breast care nurse should you need to. After you have been treated for breast cancer, you and your breast care team should agree on a care plan....Follow up care aims to detect local and regional recurrence, or a new breast cancer, but you will not usually have tests to detect a distant recurrence (metastatic breast cancer)....Further support and care:It is important that you are cared for even after your treatment has finished, and that your health and support needs continue to be met. | Quick Guide to Further Information and Support: If you have a question that is not covered by this booklet or the information sources listed below, please feel free to contact us by email at info@breastcancernow.org or by calling our Freephone Information Line 08080 100 200 and we will be happy to help. See the following pages for full contact details of each organisation. Support Emotional support andpractical advice (including someone to talk to) Breast Cancer Care Macmillan Cancer Support Breast Cancer Haven (Hereford, Leeds, London) Penny Brohn Cancer Care (Bristol) Tenovus (Cardiff) HeadStrong: hair loss support and advice sessions Breast Cancer Care Out-of-hours medical care NHS Direct or NHS 111 Lymphoedema support Lymphoedema Support Network Information Breast awareness (how to Touch Look Check your breasts for signs of breast cancer) ETC. |
| Cancer Australia | NR | non-invasive breast cancer | Ductal carcinoma in situ (DCIS) are non-invasive breast cancers that are confined to the ducts or lobules of the breast | NR | Finding support. People often feel overwhelmed, scared, anxious and upset after a diagnosis of cancer. These are all normal feelings. Having practical and emotional support during and after diagnosis and treatment for cancer is very important. Support may be available from family and friends, health professionals or special support services. | NR | NR | NR | How is breast cancer diagnosed? Diagnosis of breast cancer involves the triple test. This includes: a clinical breast examination1 imaging tests – which may include a mammogram or ultrasound taking a sample of tissue (biopsy) from the breast for examination under a microscope.1 Other tests, such as blood tests or bone scans, may be done if symptoms suggest that breast cancer has spread outside the breast.1 2 Magnetic resonance imaging (MRI) may be suggested to assess extent of disease in some cases. (General Breast Cancer Diagnosis) Treatment options Treatment and care of people with cancer is usually provided by a team of health professionals – called a multidisciplinary team. Treatment for breast cancer depends on the stage and type of the disease, the severity of symptoms and the person’s general health. Treatment usually involves surgery to remove part or all of the affected breast, and removal of one or more lymph nodes from the armpit. Breast reconstruction may be available for women who have the whole breast removed (mastectomy). Radiotherapy, chemotherapy, hormonal therapies, and/or targeted therapies, may also be used.1 Research is ongoing to find new ways to diagnose and treat different types of cancer. Some people may be offered the option of participation in a clinical trial to test new ways of treating breast cancer. | NR | In addition, State and Territory Cancer Councils provide general information about cancer as well as information on local resources and relevant support groups. The Cancer Council Helpline can be accessed from anywhere in Australia by calling 13 11 20 for the cost of a local call. More information about finding support can be found on the Cancer Australia website www.canceraustralia.gov.au or Breast Cancer Network Australia www.bcna.org.au References 1. Cancer Australia. Guide for women with early breast cancer. Cancer Australia, Surry Hills, NSW, 2012. 2. National Breast and Ovarian Cancer Centre. Guide for women with secondary breast cancer. National Breast and Ovarian Cancer Centre, Surry Hills, NSW, 2010. 3. Cancer Australia. Breast changes. http://canceraustralia.nbocc.org.au/breast-cancer/awareness/breast-changes [Accessed July 2012]. 4. Cancer Australia. Breast cancer risk. http://canceraustralia.nbocc.org.au/breast-cancer/about-breast-cancer/breast-cancer-risk [Accessed July 2012]. |
| Irish Cancer Society | NR | early form of breast cancer, pre-cancer, intra-ductal or non-invasive cancer | DCIS is a very early form of breast cancer. This means that cancer cells have formed inside the milk ducts or ‘in situ’, and have not spread outside your breast. It is also called a precancerous, intraductal or non-invasive cancer. There are a number of different types of DCIS. They can be divided into high-, intermediate-, and low-grade DCIS. This grading is based on what the cells look like under the microscope. | Treatment removes early cancer cells stopping them from becoming invasive. | Coping with breast cancer: Finding out that you have breast cancer can leave you feeling a range of emotions. Fear, shock, sadness and anger are all common feelings at this time. Although DCIS is a very early and treatable form of breast cancer, you may still feel anxious and rather negative. Remember that there are people who can support you so do not be afraid to ask for help. Further support: You might find it easier to share your feelings with someone who has had a similar experience to you. For example, Reach to Recovery is a programme set up to help and support women who have recently had a breast cancer diagnosis. | NR | NR | NR | As DCIS does not usually have any symptoms, most cases are diagnosed from a mammogram (breast X-ray). Not all microcalcifications turn out to be DCIS. To confirm a diagnosis, a biopsy (removal of a piece of breast tissue) will be taken using the mammogram to guide the radiologist to the area of microcalcification. This is called a stereotactic core biopsy. If you have symptoms such as a lump or nipple discharge, you will have a range of tests. These may include a mammogram, ultrasound and core biopsy to check for abnormal cells.Page 2: The treatment offered to you will depend on factors such as the extent of DCIS and the grade. Usually, treatment involves surgery and sometimes other follow-on treatments. • Surgery - The type of surgery could range from wide local excision to mastectomy, with or without breast reconstruction. Wide local excision means a lumpectomy, which involves removing the area of DCIS and an area of normal tissue around it. A mastectomy means your whole breast is removed.• Wire-guided localisation biopsy - This helps your surgeon pinpoint the exact area to be removed during surgery. • Sentinel lymph node biopsy - e. This is a way of checking the lymph nodes in your armpit for cancer cells • Other treatments or adjuvant treatments- Adjuvant treatments are treatments given as well as surgery to reduce the risk of DCIS coming back or an invasive cancer developing. These include radiotherapy and hormone therapy. | NR | Useful organisations and websites American Cancer Society Website: www.cancer.org Irish Nutrition & Dietetic Institute Website: www.indi.ie Irish Osteoporosis Society Website: www.osteoporosis.ie Reach to Recovery Provides practical and emotional support to women with breast cancer. National Cancer Helpline 1800 200 700 Email:helpline@irishcancer.ie Website:www.cancer.ie For more information on DCIS or for confidential advice from our cancer nurse specialists, call the National Cancer Helpline on Freefone 1800 200 700 |
| National Cancer Institute | Your health care team may include the following specialists: ■ Surgeon: This type of doctor can perform surgery. You may want to find a breast cancer surgeon. ■ Medical oncologist: A medical oncologist is a doctor who specializes in treating cancer with drugs, such as chemotherapy, hormone therapy, and targeted therapy. ■ Radiation oncologist: A radiation oncologist is a doctor who specializes in treating cancer with radiation therapy. Your health care team may also include a plastic surgeon or reconstructive surgeon, an oncology nurse, a physical therapist, a social worker, and a registered dietitian. | Non-invasive, abnormal cells | A noninvasive condition in which abnormal cells are found in the lining of a breast duct. The abnormal cells have not spread outside the duct to other tissues in the breast. In some cases, ductal carcinoma in situ may become invasive cancer and spread to other tissues, although it is not known at this time how to predict which lesions will become invasive. | NR | Heres where you can go for support: Doctors, nurses, and other members of your health care team can answer questions about treatment, working, or other activities. ■ Social workers, counselors, or members of the clergy can be helpful if you want to talk about your feelings or concerns. Often, social workers can suggest resources for financial aid, transportation, home care, or emotional support. ■ Support groups can also help. In these groups, women with breast cancer or their family members meet with other patients or their families to share what they have learned about coping with the disease and the effects of treatment. Groups may offer support in person, over the telephone, or on the Internet. You may want to talk with a member of your health care team about finding a support group. Women with breast cancer often get together in support groups, but please keep in mind that each woman is different. Ways that one woman deals with cancer may not be right for another. You may want to ask your health care provider about advice you receive from other women with breast cancer. | NR | NR | NR | (General Treatment for Breast Cancer) Diagnosis: Tests: Lab Tests with Breast Tissue: The breast tissue that was removed during your biopsy can be used in special lab tests: ■ Hormone receptor tests: Some breast cancers need hormones to grow. These cancers have hormone receptors for the hormones estrogen, progesterone, or both. HER2 test: Some breast cancers have large amounts of a protein called HER2, which helps them to grow. Staging Tests: ■ Lymph node biopsy: If cancer cells are found in a lymph node, then cancer may have spread to other lymph nodes and other places in the body. Surgeons use a method called sentinel lymph node biopsy to remove the lymph node most likely to have breast cancer cells. The method of removing more lymph nodes to check for cancer cells is called axillary dissection. ■ CT scan: An x-ray machine linked to a computer takes a series of detailed pictures of your chest or abdomen. MRI: A strong magnet linked to a computer is used to make detailed pictures of your chest, abdomen, or brain. Bone scan: The doctor injects a small amount of a radioactive substance into a blood vessel. It travels through the bloodstream and collects in the bones. PET scan: You’ll receive an injection of a small amount of radioactive sugar. The radioactive sugar gives off signals that the PET scanner picks up. Treatment: Surgery is the most common treatment for breast cancer. There are several kinds of surgery. Your surgeon can describe each kind of surgery, compare the benefits and risks, and help you decide which kind might be best for you: ■ Removing part of the breast: Breast-sparing surgery is an operation to remove the cancer and a small amount of the normal tissue that surrounds it. This is also called breast-conserving surgery. It can be a lumpectomy or a segmental mastectomy (also called a partial mastectomy). A woman usually has radiation therapy after breastsparing surgery to kill cancer cells that may remain in the breast area. Removing the whole breast: Surgery to remove the whole breast (or as much of the breast tissue as possible) is a mastectomy. In some cases, a skin-sparing mastectomy may be an option. For this approach, the surgeon removes as little skin as possible. • In total (simple) mastectomy, the surgeon removes the whole breast but not the underarm lymph nodes. • In modified radical mastectomy, the surgeon removes the whole breast and most or all of the lymph nodes under the arm. After mastectomy, you may choose to have breast reconstruction. This is plastic surgery to rebuild the shape of the breast. If you’re considering breast reconstruction, talk with a plastic surgeon before having cancer surgery. | Nutrition Eating well is important before, during, and after cancer treatment. You need the right amount of calories to maintain a good weight. You also need enough protein to keep up your strength. Eating well may help you feel better and have more energy. Follow-up Care You’ll need regular checkups (such as every 3 to 6 months) after treatment for breast cancer. Checkups help ensure that any changes in your health are noted and treated if needed. If you have any health problems between checkups, contact your doctor. Eating well may help you feel better. 20 Checkups help detect… ■ Breast cancer that comes back after treatment: Breast cancer may return in the breast or chest wall. Or, it may return in any other part of the body, such as the bones, liver, lungs, or brain. ■ Health problems that can result from cancer treatment ■ NCI’s Cancer Information Service can help you locate programs, services, and NCI publications. Call 1-800-4-CANCER (1-800-422-6237). Or, chat using LiveHelp (https://livehelp.cancer.gov), NCI’s instant messaging service. ■ Your doctor or a sex counselor may be helpful if you and your partner are concerned about the effects of breast cancer on your sex life. Ask your doctor about possible treatment of side effects and whether these effects are likely to last. Whatever the outlook, you and your partner may find it helps to discuss your concerns | Additonal Support: NCI’s Cancer Information Service can help you locate programs, services, and NCI publications. Call 1-800-4-CANCER (1-800-422-6237). Or, chat using LiveHelp (https://livehelp.cancer.gov), NCI’s instant messaging service. |
| National Cancer Institute | NR | abnormal cells, not invasive cancer | DCIS stands for ductal carcinoma in situ. If you have DCIS, this means that abnormal cells were found in the lining of the breast duct, but they have not spread outside the duct to the breast tissue. These abnormal cells are not invasive cancer, but they may become cancer. | Because doctors do not know which cases of DCIS will turn into invasive cancer and which ones will not, DCIS is treated with surgery the same as invasive cancer. | NR | What are the chances that my cancer will return in the same area? Breast-Sparing Surgery: About 10% of women (1 out of every 10) who have breast-sparing surgery along with radiation therapy get cancer in the same breast within 12 years....Mastectomy: About 5% of women (1 out of every 20) who have a mastectomy will get cancer on the same side of their chest within 12 years....Mastectomy with Reconstruction: Your chances are the same as mastectomy, since breast reconstruction surgery does not affect the chances of the cancer returning.... | NR | Breast-Sparing Surgery: 10% of women (1 out of every 10) who have breast-sparing surgery and radiation therapy get cancer in the same breast within 12 years. Mastectomy: 5% of women (1 out of every 20) who have a mastectomy will get cancer on the same side of their chest within 12 years. Mastectomy with reconstruction: Chances are the same as mastectomy, breast reconstruction surgery does not affect the chances of the cancer returning | Because doctors do not know which cases of DCIS will turn into invasive cancer and which ones will not, DCIS is treated with surgery the same as invasive cancer. Surgery choices for DCIS are based on how much of the breast has abnormal cells in it and where they are in the breast... Most women who have DCIS or breast cancer that can be treated with surgery have three surgery choices. They are: ■■ Breast-sparing surgery, followed by radiation therapy ■■ Mastectomy ■■ Mastectomy with breast reconstruction surgery...Breast-Sparing Surgery Breast-sparing surgery means the surgeon removes only the DCIS or cancer and some normal tissue around it...■■ Lumpectomy ■■ Partial mastectomy ■■ Breast-conserving surgery ■■ Segmental mastectomy...After breast-sparing surgery, most women also receive radiation therapy. The main goal of this treatment is to keep cancer from coming back in the same breast. Some women will also need chemotherapy, hormone therapy, and/or targeted therapy. | NR | Recovering from Surgery: Will I have pain after surgery?....How long before I can return to normal activities? ….What other problems might I have? …..What other types of treatment will I need? Life After Surgery: What will my breast look like after surgery?...Will my breast have feeling?....Will I need more surgery?...Will the type of surgery I have affect how long I live?...What are the chances that my cancer will return in the same area? Think about whats important to you: Thinking about these questions and talking them over with others might help. Surgery Choices ■■ If I have breast-sparing surgery, am I willing and able to have radiation therapy 5 days a week for 5 to 8 weeks? ■■ If I have a mastectomy, do I also want breast reconstruction surgery? ■■ If I have breast reconstruction surgery, do I want it at the same time as my mastectomy? ■■What treatment does my insurance cover? What do I have to pay for? Life after Surgery ■■ How important is it to me how my breast looks after cancer surgery? ■■ How important is it to me how my breast feels after cancer surgery? ■■ If I have a mastectomy and do not have reconstruction, will my insurance cover prosthesis and special bras? ■■Where can I find a breast prosthesis and special bras? Learning More ■■ Do I want a second opinion? ■■ Is there someone else I should talk with about my surgery choices? ■■What else do I want to learn or do before I make my choice about breast cancer surgery? Question List with Answers including infomration on Life After Surgery and Recovering from Surgery for three various treatment options ( Breast-Sparing Surgery, Masectomy, Masectomy with Reconstruction). Ways to Laern More Resource list (ex. National Cancer Institute We offer research-based information for patients and their families, health professionals, cancer researchers, advocates, and the public. ■■ Call: NCI’s Cancer Information Service at 1-800-4-CANCER (1-800-422-6237) ■■ Visit: www.cancer.gov or www.cancer.gov/espanol ■■ Chat: www.cancer.gov/livehelp ■■ E-mail: cancergovstaff@mail.nih.gov) |
| HealthDirect | NR | type of breast cancer, pre-malignant condition, abnormal cells, | Ductal carcinoma is a type of breast carcinoma (cancer), making up approximately 80% of all cases of breast cancer diagnosed in Australia. Ductal carcinoma may be divided into in-situ carcinoma (DCIS) and invasive ductal carcinoma (IDC). Non-invasive cancers stay within the milk ducts or lobules of the breast. They do not invade normal tissues unlike invasive breast cancers. Ductal carcinoma in situ is sometimes called a pre-malignant condition. It describes an abnormal growth of the milk duct cells. The cells share similar microscopic features with invasive breast cancer, but are confined to the milk ducts (see the image below). The term ‘in situ’ means that the cancer has not yet penetrated through the basement membrane (the membrane at the base of the epithelial lining of ducts or glands). | To prevent invasive breast cancer development. | NR | Progression of Pre-Invasive Ductal Carcinoma (Ductal Carcinoma in Situ; DCIS; Breast Cancer) The term DCIS describes an abnormal growth of cancerous cells in the milk ducts of the breast. The abnormal cells have similar features to those of invasive breast cancer, but are not able to spread beyond the milk duct into surrounding normal breast tissue. It is estimated that women who have had DCIS are 4–12 times more likely to develop subsequent invasive breast cancer despite adequate treatment. No reliable factors have been identified which predispose people with DCIS to subsequently develop invasive breast cancer but the risk may be higher when the DCIS lesion displays certain microscopic features....Women with DCIS are at increased risk of developing invasive breast cancer in the future. However, as most cases of DCIS are now treated, it is not known exactly how high this risk is, or how quickly the progression from in situ to invasive cancer is likely to occur. Local recurrence (the tumour regrowing in the same area) rates of DCIS depend on the type of treatment that is implemented. There is a 1.4% recurrence rate when DCIS is treated with both conservative breast surgery and radiotherapy. | Women with DCIS are at an increased risk of developing invasive breast cancer in the future. With most DCIS treated, it is not known how high this risk is or how quickly progression to invasive cancer may occur | Estimated that women who have had DCIS are 4-12 times more likely to develop subsequent invasive breast cancer despite treatment. There is a 1.4% reccurence rate when DCIS treated with conservative breast surgery and radiotherapy. | How is Pre-Invasive Ductal Carcinoma (Ductal Carcinoma In Situ; DCIS; Breast Cancer) Diagnosed? Initial tests: Any breast symptom, such as a lump or nipple discharge, is assessed with the ‘triple test’. This includes examination of the breast, imaging of the breast through mammography (breast xray) or ultrasound, and sampling of the breast tissue with fine needle aspiration (FNA), core biopsy or open biopsy. Further tests: Following a diagnosis of breast cancer, blood tests including full blood count and liver function tests may be used to assess the possibility of the cancer spreading to the liver or bone marrow. Other imaging tests, including chest x-ray, bone scan, positron emission tomography (PET) scan, abdominal CT or liver ultrasound may also be used if symptoms suggest that the cancer has spread. Surgical treatment: Surgical treatment can be divided into two major streams: Breast conserving surgery with complete local excision (CLE) and axillary dissection, or total mastectomy with axillary dissection (removal of axillary lymph nodes).These two procedures have been shown to confer similar survival rates at 5 and 10 years post-procedure. The decision on choice of treatment is therefore based on clinical features, such as the size and location of the tumour: Larger, widespread cancers or cancer near the nipple may require mastectomy (removal of all breast tissue). This can be followed by reconstructive surgery; Smaller tumours or tumours located in the outer quadrant in larger breasts may be removed by lumpectomy (removal of lump) or partial mastectomy (removal of selected areas of breast tissue). Radiotherapy usually follows the procedure; In selected cases, with small well-differentiated tumours, omission of axillary dissection may be considered. Radiotherapy: Radiation therapy after surgical treatment has been proven to reduce the risk of disease recurrence and development of invasive breast cancer. Studies have shown that at 15 years, the rate of developing invasive breast cancer was about half in people who had both radiation therapy and surgery as compared to those who only had surgical therapy. Systemic therapy Chemotherapy has never been investigated or used in the treatment of women with DCIS. Only tamoxifen (a drug that blocks the oestrogen receptor) which is an adjuvant (a treatment given after another treatment) hormonal treatment has been investigated in studies to determine its effectiveness in the treatment of DCIS. Hormonal therapy: Women with tumours that are proven on histology to express oestrogen receptors should be offered hormone therapies such as tamoxifen or ovarian ablation therapy (procedure whereby ovaries are intentionally damaged to decrease production of oestrogen). | NR | Reference List: Pre-Invasive Ductal Carcinoma (Ductal Carcinoma In Situ; DCIS; Breast Cancer) References 1. Australian Institute of Health and Welfare & National Breast Cancer Centre 2006. Breast cancer in Australia: an overview, 2006. Cancer series no. 34. cat. no. CAN 29. Canberra: AIHW. [cited 28 September 2015]. Available from: [URL Link] |
| Cancer Prevention and Treatment Fund | For most women with DCIS, there will be many health professionals involved in your diagnosis and treatment. You will meet some of these professionals. Others you will only hear about. The health professionals often include a: *Mammographer (breast radiologist) – looks at pictures of your breasts to see if there is anything abnormal. *Breast surgeon – performs a biopsy to find out whether or not you have DCIS. He or she also does breast surgery to remove the DCIS. * Pathologist – looks at breast tissue removed during surgery to determine if you have DCIS or another medical problem. The pathologist writes a pathology report that says whether you have DCIS or not. *Medical oncologist – provides medical care that may include hormonal therapy. The medical oncologist may also work with all your other health professionals. *Radiation oncologist – responsible for any radiation therapy after DCIS surgery. *Reconstructive plastic surgeon – can create a new breast-like shape for women who have had a mastectomy (breast removed). * patient navigator. *nurse navigator | early breast cancer | A very early breast cancer that is usually too small to form a lump. Some doctors call it a pre-cancer. DCIS is usually very small and seen on a mammogram as tiny white specks. DCIS is in the milk ducts and can not spread to other parts of your breast or body. DCIS is like a warning sign. It tells you that your chance of getting invasive breast cancer is higher than for other women. | Treatment advised as it is unclear if DCIS will become invasive or not. | NR | DCIS is like a warning sign. It tells you that your chance of getting invasive breast cancer is higher than for other women. Since no one knows for sure if your DCIS will turn into invasive cancer, you will want to get it treated. If you choose to get a lumpectomy, radiation will reduce the chances of DCIS or cancer developing later in the same breast. Hormonal treatment slightly reduces the chances of having DCIS or invasive breast cancer in either breast in the future. Raditiaon therapy, hormonal therapy and masectomy will reduce the chances of DCIS or cancer developing later in the same breast. Although a mastectomy will not help you live longer, it does lower the chances of getting breast cancer in the future. | NR | NR | Your doctor likely did a biopsy to find out if you have DCIS....Women diagnosed with DCIS almost always need surgery. The three choices are: *Lumpectomy followed by Radiation Therapy: Lumpectomy is a type of surgery in which the surgeon removes only the DCIS and some normal tissue around it. It is sometimes called “breast-sparing surgery,” “breast-conserving surgery,” or “partial mastectomy."...radiation therapy helps prevent DCIS and invasive cancer from growing in the same breast later on. *Mastecomy (Breast Removal) : Having a mastectomy (surgery to remove the entire breast) is usually not necessary for women with DCIS. But, some women choose to have a mastectomy. *Masectomy with Breast Reconstruction Surgery: If you have a mastectomy and do not want any more surgery, you can wear a prosthesis (breast-like form) in your bra. *“Modified radical” mastectomy: is a more serious surgery and is not needed. *Double (or bilateral) mastectomy is not recommended *Hormonal Treatment: slightly reduces the chances of having DCIS or invasive breast cancer in either breast in the future. However, it has side effects.*Sentinel Lymph Node Biopsy: Sometimes one or more lymph nodes under the arm (axillary lymph nodes) are also removed and checked for cancer. *Chemotherapy: Chemotherapy is almost never needed for DCIS. | Raditiaon Therapy after Lumpectomy. Breast Reconstruction after masectomy. Hormonal therapy for DCIS prevents the hormones in your body from helping cancer to develop. Stay Healthy *Have regular breast exams: Now that you have been diagnosed with DCIS, it is very important that you have regular breast exams. *Eat a healthy diet: Even women with good health habits can get DCIS. But, eating right and being active may lower your chance of getting breast cancer or DCIS again. *Stay away from cigarettes and other tobacco smoke: Smoking increases your risk of cancer and heart disease. Second-hand smoke may increase the risk of breast cancer. Learn as much as you want to know: There are many ways to learn more about DCIS... | Questions You Want to Ask Your Doctor Note Area, Words To Know/Glossary, Ways to Learn More, Support Information Group, Resources in Washington, D.C., Chart for Comparing Surgical Options (Question Prompt List) |
| Cancer Society NZ | NR | early form of breast cancer, pre-cancerous, non-invasive, intra-ductal cancer, abnormal cells | Ductal carcinoma in situ (DCIS) is an early form of breast cancer, sometimes described as pre-cancerous, non-invasive, or intraductal cancer. This means that the cancer cells are inside the milk ducts or ‘in situ’ and have not developed the ability to spread to other parts of the breast or outside it. The milk ducts are channels in the breast that carry milk to the nipple. DCIS is often split into three groups: high grade, intermediate grade and low grade. The grading of DCIS, for example high, intermediate or low, indicates how actively the abnormal cells are dividing. The grade relates to how the cells look under the microscope after a biopsy. High grade DCIS is when the cells are dividing more rapidly than low grade. Intermediate grade represents DCIS that lies between low and high grade. | Without treatment, DCIS may spread into breat tissue becoming invasive. | When you are diagnosed with DCIS, you may feel a range of emotions because the treatment is often very similar to ‘actual’ cancer. Your friends and family will often react as if you had a cancer that could spread so first early fears for your future are significant. You may find it helpful to talk these through with friends or family or another woman who has had DCIS. The Cancer Society offers a service called Cancer Connect where you can be linked by phone to talk to a woman who has had DCIS. | Not every woman with DCIS will go on to develop breast cancer if it is left untreated, but it isn’t possible to predict when DCIS will develop into breast cancer. Recurrence or return of DCIS after local excisions is uncommon. Recurrence after mastectomy is less than 1 percent. | Recurrence or return of DCIS after local excisions is uncommon. | Recurrence after masectomy in less than 1 percent. | The following describes the treatment options for DCIS: • Wide local excision only or removal of the DCIS is more commonly used for small areas of low grade DCIS. • Wide local excision followed by radiation treatment is recommended if you have high grade DCIS. • Mastectomy is recommended for women who have a large area of DCIS or several separate areas of DCIS within the breast. If mastectomy is recommended you may like to discuss breast reconstruction with your surgeon. If, after removing microcalcification, invasive cancer is found, your surgeon may then advise Lymph Node Removal. Hormone Treatment. | Follow-up treatment. All women treated for DCIS should have long-term, regular clinical examinations and mammography. Most recurrences of DCIS are picked up by mammography. | Suggested Websites: National Breast and Ovarian Cancer Centre (Australia) http://www.understanding ductal carcinoma in situ (DCIS) and deciding about treatment-communication aid BreastScreen Aotearoa www.nsu.govt.nz cancer information hotline; Contact your local Cancer Society, telephone 0800 CANCER (226 237) to speak confidentially with a cancer information nurse |
