# Supplementary material for: Quality of DCIS information on the internet: a content analysis
Source: Breast Cancer Res Treat. 2019 Jun 18;177(2):295–305. doi: 10.1007/s10549-019-05315-8 (PMC6661062; doi:10.1007/s10549-019-05315-8)
Supplement: Supplementary file 3 — Supplementary material 3 (DOCX 18 kb) [file 10549_2019_5315_MOESM3_ESM.docx]

Supplementary File 3. Labels for DCIS in included tools

| Organization  (country, date) | Labels used | | | | | | | | Total labels used (n) |
| --- | --- | --- | --- | --- | --- | --- | --- | --- | --- |
|  | Stage 0 cancer | Pre-cancer or pre-invasive cancer | Early form of breast cancer | Non-invasive breast cancer | Abnormal cells | Cancer cells | Not breast cancer | DCIS only |  |
| Total resources featuring specific labels (n) | 9 (23.1) | 14  (35.9) | 13  (33.3) | 29  (74.4) | 14  (35.9) | 6  (15.4) | 0  (0.0) | 2  (5.1) |  |
| Breast Cancer Care UK  England  2018 [39] | X | X | X | X |  |  |  |  | 4 |
| Cancer Council Western Australia  Australia  2018 [40] |  |  |  | X | x |  |  |  | 2 |
| Dr. Susan Love Research Foundation  United States  2018 [41] |  | X |  | x | x |  |  |  | 3 |
| National Comprehensive Cancer Center  United States  2018 [42] | X |  |  | x |  |  |  |  | 2 |
| National Health Service  Scotland  2018 [43] | x |  |  | x |  |  |  |  | 2 |
| Cancer Care Nova Scotia  Canada  2018 [44] |  |  |  |  |  |  |  | x | 1 |
| Susan G. Komen  United States  2018 [45] |  | x |  | x | x |  |  |  | 3 |
| Susan G. Komen  United States  2018 [46] |  | x |  | x |  |  |  |  | 2 |
| Ohio State University Comprehensive Cancer Center  United States  2018 [47] | x |  |  | x | x | x |  |  | 4 |
| The Pennine Acute Hospitals  England  2018 [48] |  | x | x | x |  |  |  |  | 3 |
| University of Iowa Hospitals and Clinics  United States  2018 [49] | x |  |  | x |  |  |  |  | 2 |
| American Society of Clinical Oncology  United States  2017 [50] |  |  |  | x |  |  |  |  | 1 |
| BreastCancer.org  United States  2017 [51] |  |  |  | x |  |  |  |  | 1 |
| Breast Screen Aotearoa  New Zealand  2017 [52] |  |  | x | x |  |  |  |  | 2 |
| Cancer Australia  Australia  2017 [53] |  |  |  | x | x |  |  |  | 2 |
| Cancer Research UK  England  2017 [54] |  |  |  |  | x |  |  |  | 1 |
| Cancer Treatment Centers of America  United States  2017 [55] |  |  |  |  | x |  |  |  | 1 |
| Health Talk.org, University of Oxford and DIPEx  England  2017 [56] |  | x | x | x | x | x |  |  | 5 |
| National Health Service  England  2017 [57] |  |  |  | x |  | x |  |  | 2 |
| Alaska Breast Care and Surgery  United States  2016[ 58] | x | x |  |  |  |  |  |  | 2 |
| American Cancer Society  United States  2016 [59] |  | x |  | x |  | x |  |  | 3 |
| California Department of Health Care Services  United States  2016 [60] |  |  |  | x |  |  |  |  | 1 |
| Living Beyond Breast Cancer  United States  2016 [61] | x |  |  |  |  | x |  |  | 2 |
| National Health Service  England  2016 [62] |  |  |  | x |  |  |  |  | 1 |
| Worcester Breast Surgery  England  2016 [63] |  | x | x | x |  |  |  |  | 3 |
| The Newcastle upon Tyne Hospitals  England  2016 [64] |  |  |  |  |  |  |  | x | 1 |
| Princess Margaret Hospital – University Health Network  Canada  2016 [65] |  |  | x |  |  |  |  |  | 1 |
| American Cancer Society  United States  2015 [66] | x |  | x | x | x |  |  |  | 4 |
| Macmillan Cancer Support  England  2015 [67] | X |  | X | X |  |  |  |  | 3 |
| Westmead Breast Cancer Institute  Australia  2015 [68] |  | x | x |  |  |  |  |  | 2 |
| Breast Cancer Action  United States  2014 [69] |  | x |  | x | x |  |  |  | 3 |
| Breast Cancer Now  England  2013 [70] |  |  | x | x |  |  |  |  | 2 |
| Cancer Australia  Australia  2013 [71] |  |  |  | x |  |  |  |  | 1 |
| Irish Cancer Society  Ireland  2013 [72] |  | x | x | x |  |  |  |  | 3 |
| National Cancer Institute  United States  2012 [73] |  |  |  | x | x |  |  |  | 2 |
| National Cancer Institute  United States  2012 [74] |  |  |  | x | x |  |  |  | 2 |
| HealthDirect  Australia  2012 [75] |  | x |  |  | x | x |  |  | 3 |
| Cancer Prevention and Treatment Fund  United States  2011 [76] |  |  | x |  |  |  |  |  | 1 |
| Cancer Society NZ  New Zealand  2011 [77] |  | x | x | x | x |  |  |  | 4 |
