# Supplementary material for: Quality of DCIS information on the internet: a content analysis
Source: Breast Cancer Res Treat. 2019 Jun 18;177(2):295–305. doi: 10.1007/s10549-019-05315-8 (PMC6661062; doi:10.1007/s10549-019-05315-8)
Supplement: Supplementary file 4 — Supplementary material 4 (DOCX 16 kb) [file 10549_2019_5315_MOESM4_ESM.docx]

Supplementary File 4. DCIS information tool content by PCC domain[8]

| Organization  (country, date) | PCC domains | | | | | | PCC domains  included (n) |
| --- | --- | --- | --- | --- | --- | --- | --- |
|  | Fostering the relationship | Exchanging information | Addressing emotions | Managing uncertainty | Making decisions | Enabling self-management |  |
|  | Name specialties involved in DCIS care | Describe DCIS, distinguish from invasive cancer | Acknowledge concerns, offer coping strategies | Risk of progression and recurrence | Treatment options, factors affecting choice | Follow-up and self-care strategies |  |
| Total resources featuring specific PCC domains (n) | 11 (28.2) | 35 (89.7) | 11 (28.2) | 31 (79.5) | 39 (100.0) | 18 (46.2) |  |
| Breast Cancer Care UK  England  2018 [39] | --- | X | X | X | X | X | 5 |
| Cancer Council Western Australia  Australia  2018 [40] | X | X | --- | X | X | --- | 4 |
| Dr. Susan Love Research Foundation  United States  2018 [41] | --- | X | --- | X | X | --- | 3 |
| National Comprehensive Cancer Center  United States  2018 [42] | X | X | --- | X | X | X | 5 |
| National Health Service  Scotland  2018 [43] | X | X | --- | X | X | X | 4 |
| Cancer Care Nova Scotia  Canada  2018 [44] | --- | --- | X | --- | X | X | 3 |
| Susan G. Komen  United States  2018 [45] | --- | X | --- | X | X | --- | 3 |
| Susan G. Komen  United States  2018 [46] | --- | X | --- | X | X | --- | 3 |
| Ohio State University Comprehensive Cancer Center  United States  2018 [47] | --- | X | --- | X | X | X | 4 |
| The Pennine Acute Hospitals  England  2018 [48] | X | X | --- | X | X | X | 5 |
| University of Iowa Hospitals and Clinics  United States  2018 [49] | X | X | --- | X | X | --- | 4 |
| American Society of Clinical Oncology  United States  2017 [50] | --- | --- | --- | X | X | --- | 2 |
| BreastCancer.org  United States  2017 [51] | --- | X | --- | X | X | X | 4 |
| Breast Screen Aotearoa  New Zealand  2017 [52] | --- | X | --- | X | X | X | 4 |
| Cancer Australia  Australia  2017 [53] | --- | X | --- | X | X | --- | 3 |
| Cancer Research UK  England  2017 [54] | --- | X | --- | X | X | X | 4 |
| Cancer Treatment Centers of America  United States  2017 [55] | --- | X | --- | X | X | --- | 3 |
| Health Talk.org, University of Oxford and DIPEx  England  2017 [56] | --- | X | X | --- | X | --- | 3 |
| National Health Service  England  2017 [57] | --- | X | --- | X | X | --- | 3 |
| Alaska Breast Care and Surgery  United States  2016[ 58] | --- | X | --- | --- | X | --- | 2 |
| American Cancer Society  United States  2016 [59] | --- | X | --- | X | X | --- | 3 |
| California Department of Health Care Services  United States  2016 [60] | X | X | --- | X | X | X | 5 |
| Living Beyond Breast Cancer  United States  2016 [61] | X | X | X | X | X | --- | 5 |
| National Health Service  England  2016 [62] | --- | X | X | --- | X | --- | 3 |
| Worcester Breast Surgery  England  2016 [63] | --- | X | --- | X | X | --- | 3 |
| The Newcastle upon Tyne Hospitals  England  2016 [64] | --- | --- | X | --- | X | X | 3 |
| Princess Margaret Hospital – University Health Network  Canada  2016 [65] | --- | X | --- | X | X | X | 4 |
| American Cancer Society  United States  2015 [66] | --- | X | --- | X | X | --- | 3 |
| Macmillan Cancer Support  England  2015 [67] | X | X | --- | X | X | X | 5 |
| Westmead Breast Cancer Institute  Australia  2015 [68] | --- | X | X | X | X | --- | 4 |
| Breast Cancer Action  United States  2014 [69] | --- | X | --- | X | X | --- | 3 |
| Breast Cancer Now  England  2013 [70] | X | X | --- | X | X | X | 5 |
| Cancer Australia  Australia  2013 [71] | --- | X | X | --- | X | --- | 3 |
| Irish Cancer Society  Ireland  2013 [72] | --- | X | X | --- | X | --- | 3 |
| National Cancer Institute  United States  2012 [73] | X | X | X | --- | X | X | 5 |
| National Cancer Institute  United States  2012 [74] | --- | X | --- | X | X | --- | 3 |
| HealthDirect  Australia  2012 [75] | --- | X | --- | X | X | X | 4 |
| Cancer Prevention and Treatment Fund  United States  2011 [76] | X | X | --- | X | X | X | 5 |
| Cancer Society NZ  New Zealand  2011 [77] | --- | X | X | X | X | X | 5 |
