# Supplementary material for: Quality of DCIS information on the internet: a content analysis
Source: Breast Cancer Res Treat. 2019 Jun 18;177(2):295–305. doi: 10.1007/s10549-019-05315-8 (PMC6661062; doi:10.1007/s10549-019-05315-8)
Supplement: Supplementary file 5 — Supplementary material 5 (DOCX 19 kb) [file 10549_2019_5315_MOESM5_ESM.docx]

Supplementary File 5. DCIS information tools assessed for quality with DISCERN instrument [38]

| Source  (Organization, country, date published) | Q1 | Q2 | Q3 | Q4 | Q5 | Q6 | Q7 | Q8 | Q9 | Q10 | Q11 | Q12 | Q13 | Q14 | Q15 | Q16 (Overall) |
| --- | --- | --- | --- | --- | --- | --- | --- | --- | --- | --- | --- | --- | --- | --- | --- | --- |
| Breast Cancer Care UK  England  2018 [39] | Y | P | Y | P | Y | P | N | Y | Y | N | N | Y | N | Y | P | Moderate |
| Cancer Council Western Australia  Australia  2018 [40] | Y | Y | Y | P | Y | Y | Y | Y | Y | N | P | N | Y | Y | Y | High |
| Dr. Susan Love Research Foundation  United States  2018 [41] | P | P | Y | P | P | P | Y | Y | Y | N | N | N | N | Y | N | Moderate |
| National Comprehensive Cancer Center  United States  2018 [42] | Y | Y | Y | P | Y | Y | Y | Y | Y | N | P | N | N | Y | Y | High |
| National Health Service  Scotland  2018 [43] | N | N | Y | P | Y | P | Y | Y | Y | N | P | N | Y | Y | N | Moderate |
| Cancer Care Nova Scotia  Canada  2018 [44] | N | N | Y | P | Y | N | N | P | P | N | P | N | Y | N | N | Low |
| Susan G. Komen  United States  2018 [45] | N | N | Y | P | Y | P | P | Y | P | N | N | Y | N | P | N | Moderate |
| Susan G. Komen  United States  2018 [46] | N | N | Y | P | Y | P | P | Y | P | N | N | Y | N | P | Y | Moderate |
| Ohio State University Comprehensive Cancer Center  United States  2018 [47] | N | N | Y | P | Y | P | N | Y | P | N | P | N | N | Y | N | Low |
| The Pennine Acute Hospitals  England  2018 [48] | Y | Y | Y | P | Y | Y | Y | Y | Y | N | Y | P | Y | P | N | High |
| University of Iowa Hospitals and Clinics  United States  2018 [49] | N | N | Y | P | Y | P | P | P | Y | N | N | N | N | P | N | Low |
| American Society of Clinical Oncology  United States  2017 [50] | N | N | Y | P | Y | P | N | Y | P | N | N | N | N | P | Y | Low |
| BreastCancer.org  United States  2017 [51] | N | N | Y | P | Y | P | N | Y | Y | P | N | N | N | Y | N | Moderate |
| Breast Screen Aotearoa  New Zealand  2017 [52] | P | N | Y | N | Y | N | N | N | P | N | N | P | N | Y | N | Low |
| Cancer Australia  Australia  2017 [53] | N | N | P | P | Y | P | P | Y | Y | N | N | Y | N | Y | N | Moderate |
| Cancer Research UK  England  2017 [54] | P | P | Y | P | Y | P | P | Y | Y | N | N | Y | N | P | N | Moderate |
| Cancer Treatment Centers of America  United States  2017 [55] | N | N | Y | P | Y | N | N | N | P | P | N | N | N | P | N | Low |
| Health Talk.org, University of Oxford and DIPEx  England  2017 [56] | N | N | Y | P | Y | Y | Y | Y | Y | N | N | Y | Y | Y | Y | High |
| National Health Service  England  2017 [57] | Y | P | Y | P | Y | P | P | Y | P | P | N | N | N | P | N | Moderate |
| Alaska Breast Care and Surgery  United States  2016[ 58] | N | N | P | P | Y | N | N | N | P | N | N | N | N | P | N | Low |
| American Cancer Society  United States  2016 [59] | N | N | Y | Y | Y | P | N | Y | P | N | N | N | N | P | N | Low |
| California Department of Health Care Services  United States  2016 [60] | P | Y | Y | P | Y | Y | Y | Y | Y | N | Y | N | N | P | Y | High |
| Living Beyond Breast Cancer  United States  2016 [61] | Y | Y | Y | P | Y | Y | Y | Y | Y | N | P | N | Y | Y | Y | High |
| National Health Service  England  2016 [62] | N | N | Y | P | Y | P | Y | Y | Y | N | P | N | Y | Y | N | Moderate |
| Worcester Breast Surgery  England  2016 [63] | N | N | Y | P | Y | P | N | N | Y | N | N | N | N | Y | N | Low |
| The Newcastle upon Tyne Hospitals  England  2016 [64] | Y | P | P | P | Y | N | P | Y | P | N | P | N | N | P | N | Moderate |
| Princess Margaret Hospital – University Health Network  Canada  2016 [65] | Y | P | Y | P | Y | P | N | Y | Y | N | N | N | N | Y | N | Moderate |
| American Cancer Society  United States  2015 [66] | N | N | N | Y | Y | P | P | Y | Y | N | N | Y | N | Y | N | Moderate |
| Macmillan Cancer Support  England  2015 [67] | Y | Y | Y | Y | Y | Y | Y | Y | Y | Y | P | Y | Y | Y | P | High |
| Westmead Breast Cancer Institute  Australia  2015 [68] | N | N | Y | P | Y | P | N | Y | Y | Y | P | Y | N | P | N | Moderate |
| Breast Cancer Action  United States  2014 [69] | Y | Y | Y | Y | Y | Y | N | Y | Y | N | P | Y | N | Y | Y | High |
| Breast Cancer Now  England  2013 [70] | Y | Y | Y | P | Y | P | Y | Y | Y | Y | P | N | P | Y | P | High |
| Cancer Australia  Australia  2013 [71] | N | N | P | Y | Y | P | P | Y | P | N | N | N | N | P | N | Moderate |
| Irish Cancer Society  Ireland  2013 [72] | Y | P | Y | P | Y | P | Y | Y | P | N | N | P | N | Y | N | Moderate |
| National Cancer Institute  United States  2012 [73] | Y | Y | Y | P | Y | P | P | Y | Y | N | N | N | P | Y | Y | Moderate |
| National Cancer Institute  United States  2012 [74] | Y | Y | Y | Y | Y | P | Y | Y | P | N | P | N | N | Y | N | Moderate |
| HealthDirect  Australia  2012 [75] | N | N | P | P | N | P | Y | P | N | N | N | N | N | P | N | Low |
| Cancer Prevention and Treatment Fund  United States  2011 [76] | Y | Y | Y | Y | Y | P | Y | Y | Y | Y | P | N | N | Y | P | High |
| Cancer Society NZ  New Zealand  2011 [77] | Y | P | Y | P | Y | P | Y | Y | P | N | N | Y | N | P | N | Moderate |

Y yes, N no, P partial
